# Dystrophin-deficient dogs with reduced myostatin have unequal muscle growth and greater joint contractures

**DOI:** 10.1186/s13395-016-0085-7

**Published:** 2016-04-04

**Authors:** Joe N. Kornegay, Daniel J. Bogan, Janet R. Bogan, Jennifer L. Dow, Jiahui Wang, Zheng Fan, Naili Liu, Leigh C. Warsing, Robert W. Grange, Mihye Ahn, Cynthia J. Balog-Alvarez, Steven W. Cotten, Monte S. Willis, Candice Brinkmeyer-Langford, Hongtu Zhu, Joe Palandra, Carl A. Morris, Martin A. Styner, Kathryn R. Wagner

**Affiliations:** Department of Pathology and Laboratory Medicine, University of North Carolina at Chapel Hill, Chapel Hill, NC 27599 USA; Department of Neurology, University of North Carolina at Chapel Hill, Chapel Hill, NC 27599 USA; Department of Psychiatry, University of North Carolina at Chapel Hill, Chapel Hill, NC 27599 USA; Department of Biostatistics, University of North Carolina at Chapel Hill, Chapel Hill, NC 27599 USA; Department of Computer Science, University of North Carolina at Chapel Hill, Chapel Hill, NC 27599 USA; Department of Veterinary Integrative Biosciences, College of Veterinary Medicine and Biomedical Sciences, Texas A&M University, College Station, TX 77843-4458 USA; The Hugo W. Moser Research Institute at Kennedy Krieger Institute and Departments of Neurology and Neuroscience, Johns Hopkins School of Medicine, Baltimore, MD 21205 USA; Department of Human Nutrition, Foods and Exercise, Virginia Tech University, Blacksburg, VA 24061 USA; Department of Pathology, The Ohio State University, Columbus, OH 43210 USA; Rare Disease Research Unit, Pfizer, Inc., Cambridge Park Drive, Cambridge, MA USA

**Keywords:** Muscular dystrophy, Myostatin inhibition, Dogs, Golden retriever muscular dystrophy (GRMD), Whippets, Muscle hypertrophy, Contractures

## Abstract

**Background:**

Myostatin (*Mstn*) is a negative regulator of muscle growth whose inhibition promotes muscle growth and regeneration. Dystrophin-deficient mdx mice in which myostatin is knocked out or inhibited postnatally have a less severe phenotype with greater total mass and strength and less fibrosis and fatty replacement of muscles than mdx mice with wild-type myostatin expression. Dogs with golden retriever muscular dystrophy (GRMD) have previously been noted to have increased muscle mass and reduced fibrosis after systemic postnatal myostatin inhibition. Based partly on these results, myostatin inhibitors are in development for use in human muscular dystrophies. However, persisting concerns regarding the effects of long-term and profound myostatin inhibition will not be easily or imminently answered in clinical trials.

**Methods:**

To address these concerns, we developed a canine (*GRippet*) model by crossbreeding dystrophin-deficient GRMD dogs with *Mstn*-heterozygous (*Mstn*^+/−^) whippets. A total of four *GRippets* (dystrophic and *Mstn*^+/−^), three GRMD (dystrophic and *Mstn* wild-type) dogs, and three non-dystrophic controls from two litters were evaluated.

**Results:**

Myostatin messenger ribonucleic acid (mRNA) and protein levels were downregulated in both GRMD and *GRippet* dogs. *GRippets* had more severe postural changes and larger (more restricted) maximal joint flexion angles, apparently due to further exaggeration of disproportionate effects on muscle size. Flexors such as the cranial sartorius were more hypertrophied on magnetic resonance imaging (MRI) in the *GRippets*, while extensors, including the quadriceps femoris, underwent greater atrophy. Myostatin protein levels negatively correlated with relative cranial sartorius muscle cross-sectional area on MRI, supporting a role in disproportionate muscle size. Activin receptor type IIB (ActRIIB) expression was higher in dystrophic versus control dogs, consistent with physiologic feedback between myostatin and ActRIIB. However, there was no differential expression between GRMD and *GRippet* dogs. Satellite cell exhaustion was not observed in *GRippets* up to 3 years of age.

**Conclusions:**

Partial myostatin loss may exaggerate selective muscle hypertrophy or atrophy/hypoplasia in GRMD dogs and worsen contractures. While muscle imbalance is not a feature of myostatin inhibition in mdx mice, findings in a larger animal model could translate to human experience with myostatin inhibitors.

**Electronic supplementary material:**

The online version of this article (doi:10.1186/s13395-016-0085-7) contains supplementary material, which is available to authorized users.

## Background

Duchenne muscular dystrophy (DMD) affects approximately 1 in 5000 newborn boys in whom *DMD* gene mutations lead to progressive degeneration of cardiac and skeletal muscle [1–4]. One strategy for promoting muscle regeneration involves inhibiting myostatin (*Mstn*; growth and differentiation factor 8 (GDF-8)), a negative regulator of muscle growth [5–8]. Humans [9], cattle [10], sheep [11], and dogs [12] with *Mstn* gene mutations have dramatic muscle hypertrophy. Dystrophin-deficient mdx mice in which myostatin is knocked out (*Mstn*^*−/−*^) [13] or inhibited postnatally [14, 15] also have a less severe phenotype with greater absolute force and less fibrosis of individual muscles.

Based on these findings, there has been increasing interest in treatments to inhibit myostatin and thus promote muscle growth [16, 17]. However, other studies have identified potential negative consequences to myostatin inhibition in skeletal muscle. Muscle tendons of *Mstn*^*−/−*^ mice are hypocellular and more brittle [18]. There are differential muscle effects in myostatin-null mice with, for example, the fast-twitch predominant extensor digital longus (EDL) muscle demonstrating reduced specific isometric force (force normalized by cross-sectional area (CSA)) and greater eccentric contraction decrement compared to the slow twitch soleus [19–21]. Elimination of myostatin in the dy(W) laminin alpha2-deficient murine model of congenital muscular dystrophy was associated with increased pre-weaning mortality, potentially due to reduced fat formation [22], while blockade of myostatin with transgenic expression of follistatin in the Dyf^−/−^ model of LGMD2B and Myoshi myopathy exacerbated muscle degeneration with aging [23]. Additional questions have been raised about potential exhaustion of the pool of muscle progenitor cells (i.e., satellite cells) undergoing multiple divisions in the absence of myostatin in muscular dystrophy [24].

While genetically engineered mice have provided an extremely powerful tool to study the molecular pathogenesis of disease [25, 26], results do not necessarily extrapolate to humans, presumably due to differences between murine and human size and physiology [27]. These shortcomings are partially countered with canine models, which have been used extensively to study disease pathogenesis and treatment efficacy [28, 29]. This trend towards the use of dogs as models will likely accelerate with the recent sequencing of the canine genome [30]. We, and others, have investigated the potential therapeutic role of myostatin inhibition in dogs. Adeno-associated virus (AAV8)-mediated over expression of the inhibitory myostatin propeptide was shown to enhance muscle growth in normal dogs [31]. Analogous results were demonstrated in dystrophin-deficient golden retriever muscular dystrophy (GRMD) dogs [32]. After 13 months, treated GRMD dogs had increased muscle weights, ranging from 49 % (tibialis cranialis) to 27 % (EDL), and a suggestion of reduced fibrosis by histochemistry.

To further study the effects of prolonged loss of myostatin in a large animal model of DMD, we developed a myostatin-deficient GRMD (*GRippet*) model by crossbreeding GRMD dogs with *Mstn*-heterozygous (*Mstn*^*+/−*^) whippets [12]. *GRippet* and *Mstn*^*+/+*^ wild-type GRMD dogs, together with non-dystrophic control littermates, were assessed with functional tests, magnetic resonance imaging (MRI), and molecular/pathologic studies.

## Methods

### Animals

Dogs from a colony at the University of North Carolina at Chapel Hill (UNC-CH) received care and were assessed according to principles outlined in the National Research Council Guide for the Care and Use of Laboratory Animals. Studies were approved by the UNC-CH Institutional Animal Care and Use Committee (IACUC) through two protocols, UNC IACUC 08-103, *Cross Breeding of Muscular Dystrophy and Myostatin-Null Dogs*, and UNC IACUC 09-351, *Standard Operating Procedures—Canine X-Linked Muscular Dystrophy*. Independent of myostatin status, the dystrophic phenotype was determined based on elevation of serum creatine kinase (CK) and characteristic clinical signs. Genotype was confirmed by polymerase chain reaction (PCR) if CK results were ambiguous (Table 1).

**Table 1:** GRMD-myostatin status

Dog name	Gender	GRMD status	Myostatin status
Litter 1			
*Racer*	Male	Normal	Normal
*Dash*	Male	Affected	Heterozygote
*Flash*	Male	Affected	Normal
Litter 2			
*Endora*	Female	Carrier	Normal
*Esmeralda*	Female	Carrier	Heterozygote
*Samantha*	Female	Affected	Normal
*Tabitha*	Female	Affected	Heterozygote
*Hagatha*	Female	Affected	Normal
*Derwood*	Male	Affected	Heterozygote
*Abner*	Male	Affected	Heterozygote

#### GRMD

All GRMD colonies worldwide have been derived from the same founder dog [33]. This dog was initially used to establish a colony at Cornell University [34]. The original dystrophic golden retriever was crossed with other breeds. Accordingly, GRMD is not a disease of purebred golden retriever dogs. We use the term GRMD to refer to dogs carrying the underlying splice site mutation [35, 36].

#### *GRippets*

Whippet dogs homozygous for the myostatin-null allele *(Mstn*^*−/−*^) (so called *bully whippets)* have gross enlargement of muscle, while those that are heterozygous *(Mstn*^*+/−*^) have intermediate muscle mass and are significantly faster than their myostatin wild-type counterparts [12]. Semen from a sire *(Mstn*^*+/−*^) of *bully whippet* dogs *(Mstn*^*−/−*^) was collected for subsequent artificial insemination of a GRMD carrier to produce a first litter. The second litter was produced by breeding a GRMD male to *Speedy*, a GRMD/*Mstn*^*+/−*^ carrier from the first litter. A total of 10 dogs were studied. Buccal swabs from all dogs of the two litters were analyzed (DDC Veterinary, Fairfield, OH) to demonstrate the same *Mstn gene* deletion at nucleotides 939 and 940 described previously [12].

### Molecular tests

Prior to muscle biopsy and phenotypic tests, dogs were premedicated with acepromazine maleate (0.02 mg/kg), butorphanol (0.4 mg/kg), and atropine sulfate (0.04 mg/kg), masked, and then intubated and maintained with sevoflurane. Samples from the cranial sartorius (CS), vastus lateralis (VL), long digital extensor (LDE), and lateral head of the gastrocnemius (LHG) muscles were removed surgically via open biopsy at 8–9 months of age, snap frozen in isopentane cooled in liquid nitrogen, and stored at −80 °C.

#### RNA isolation

Total cellular ribonucleic acid (RNA) was isolated from frozen skeletal muscle with Tripure reagent (Roche, Indianapolis, IN, USA) and DNase treated with deoxyribonucleic acid (DNA)-free kit (Applied Biosystems, Foster City, CA, USA). The RNA concentrations of the individual samples were measured using a Nanodrop 2000 spectrophotometer and assessed for quality using a 2100 Bioanalyzer (Agilent Technologies, Santa Clara, CA, USA).

#### Sequencing

The canine myostatin gene was sequenced using primers that flank the 939–940 bp mutation site: F:GTGCTGTCGTTACCCTCTAA/R: GAGACATCTTTGTGGGAGTACAG (840–1040 bp). The 200 bp PCR product was cloned into plasmid cloning vector PCR2.1 with TA Cloning Kit (Invitrogen, Carlsbad, CA, USA), plasmid DNA was prepared using QIAprep Spin Miniprep Kit (Qiagen, Hilden, Germany), and the plasmid DNA was sequenced with M13 forward primer. Multiple colonies from each dog were selected, and the sequenced plasmids were assessed to demonstrate the 939–940 bp deletion in heterozygous dogs.

#### qRT-PCR analysis

Myostatin gene expression in the CS and the VL from these 10 dogs was reported previously [37]. Gene expression in these muscles plus the LDE and LHG was assessed for this current study. Quantitative realtime RT-PCR (qRT-PCR) primers were designed using Primer Express 3.0 software (Applied Biosystems, Foster City, CA, USA) for the normalizer gene hypoxanthine phosphoribosyltransferase 1 (HPRT1). The recommended ABI TaqMan Gene Expression Assay primer pair and probe were purchased for the canine *Mstn* gene (Applied Biosystems; catalog number cf02704228_m1). Samples of skeletal muscle RNA (100 ng) were reverse transcribed with oligo-dT and random octamer primers and Superscript II (Invitrogen). qRT-PCR was performed in duplicate reactions with Taqman Gene Expression PCR Master Mix on a 7900HT Fast Real-Time PCR System (Applied Biosystems).

### Phenotypic tests

For all procedures, dogs were anesthetized (above) and tests were performed as previously described [36, 38].

#### Joint angles

Pelvic limb joint angles were measured at maximal flexion and extension, with additional calculation of the range of motion (ROM), using the method of Jaegger et al. [39]. Our group had previously measured tibiotarsal joint (TTJ) angle in GRMD dogs with a different method in the context of serial peroneus longus force measurements [40, 41]. We continue to make this measurement to compare results to historical values. For this *original* TTJ angle measurement [40, 41], dogs were placed in dorsal recumbence, with the stifle held at a 90° angle and the tibia/fibula positioned parallel to the table. The angle formed by the flexor surface of the tarsus was measured using a goniometer centered over the lateral malleolus of the fibula. Values for normal dogs using this method approximate but are somewhat less than those recorded at maximal extension.

#### Tibiotarsal joint force and ECD

Force and eccentric contraction decrement (ECD) were assessed as previously described [38, 42, 43]. Briefly, TTJ flexion and extension torque was measured using a rapid-response servomotor/force transducer (model 310B LR, Aurora Scientific, Inc., Aurora, Ontario, Canada) controlled by a personal computer using custom LabView software. Supramaximal 150 V, 100-μs pulses were applied percutaneously (Model S48 Solid State Square Wave Stimulator, Grass Instruments, Quincy, MA, USA) in a 1.5-s tetanic run of 75 pulses (50/s) to the peroneal (flexion) and tibial (extension) nerves. The site of contact for the paw with the lever (moment arm) was estimated to be 75 % of the distance between the point of the hock and the distal digit. Torque (Newton (N)-meters (m)) was divided by the moment arm (m) to convert to force (N).

Eccentric contractions were induced by percutaneously stimulating the peroneal nerve using square wave pulses of 100-μs duration in a tetanic run for 1 s at a frequency of 50 Hz while simultaneously extending the TTJ with a servomotor (Aurora Scientific) [38, 43]. Thus, the muscles of the cranial tibial compartment were repeatedly stretched to induce mechanical damage. Three sets of 10 stretches for a total of 30, each set separated by 4 min, were performed. Contraction-induced injury was quantified by the force (torque) deficit (Fd) using the following equation: Fd = (Maximal isometric tetanic force [Po] before stretch − Po after stretch/Po before stretch) × 100.

#### Cranial sartorius circumference

We have previously shown that the CS muscle undergoes dramatic hypertrophy in GRMD dogs and that this hypertrophy tracks with postural abnormalities [44]. Accordingly, as previously described [38], we used CS circumference measured at surgery during routine biopsy as a surrogate for muscle hypertrophy and associated postural changes in GRMD.

#### MRI

Studies were done on either a Siemens 3T Allegra Head-Only MRI scanner using a circular polarization (CP) head coil or on a whole body MAGNETOM Trio with Tim system with a 32-channel body coil (composed of a 16 element anterior part and 16 element posterior part) at the UNC-CH Biomedical Research Imaging Center [45, 46]. Dogs were placed on an MRI gantry in the sternal (prone) position with the pelvic limbs extended and scanned using a published protocol [45]. T_2_-weighted image sequences with fat saturation (T_2_fs) and without fat saturation (T_2_w) were acquired using a variable-flip-angle turbo spin echo (TSE) sequence. A multi-spin-echo T2 (MSE-T_2_), with a ten-echo Carr-Purcell-Meiboom-Gill sequence, was acquired to calculate T2 mapping [45, 47]. The T2 mapping was calculated on a voxel-by-voxel basis for the proximal pelvic limbs of dogs in the transverse plane of MSE-T_2_ images by fitting an exponential decay curve to the signal intensity of the corresponding voxels using a linear-least-squares curve-fitting algorithm [45].

The major proximal pelvic limb muscles in T_2_w were registered and then manually segmented, while T_2_fs was used to identify the contour of each muscle. All proximal pelvic limb muscles were segmented but only five slices at the mid-femur were analyzed/quantified [48]. The T_2_fs and T2 mapping were subsequently automatically aligned to the coordinate space of T_2_w using a rigid image registration technique [45]. Thus, the muscle segmentation in T_2_w could be used in T2 mapping for the biomarker analysis. Texture analysis features were assessed as potential markers of patchy lesions such as necrosis [49, 50].

Femur length was used to correct absolute muscle volumes as previously described [46]. To determine overall scores for T2 and the texture features in each group, the proportional muscle volume was considered, so as to calculate a weighted average.

#### Muscle histochemical and immunoblot studies

Morphometric analysis of muscle was performed in a blinded fashion. Muscle fibers were aligned for true cross sections (10 μm), stained with hematoxylin and eosin (H&E), and viewed with a light microscope (Axiocam System, Zeiss; GERMANY) at high power. Numbers per 100 myofibers were counted for degenerating fibers (hyaline and/or fragmenting fibers with/without evidence of myophagocytosis), regenerating fibers (small fibers with basophilic nuclei and prominent nuclei/nucleoli), and centrally nucleated fibers (fibers with nuclei located away from the sarcolemma within the cytoplasm; CNF). Muscle fiber CSAs were determined using Scio Image Software (National Institutes of Health (NIH)). A total of 200–600 fibers were measured for each muscle of each dog.

Immunoblots of cell lysates and tissue homogenates were performed as previously described [51]. Hydroxyproline (HP) content of various muscles was measured using a modification of Woessner and colleagues [52] as described [51]. Values are expressed as μg HP/mg of total muscle protein. For activin receptor type IIB (ActRIIB) Western blots, frozen muscle samples were homogenized in Tissue Extraction Reagent I (Invitrogen), followed by quantification with the BCA Protein Assay Kit (ThermoFisher Scientific, Grand Island, NY, USA). Five micrograms (μg) of total protein from each muscle were loaded onto Novex 10 % Tris-Glycine gels and transferred to polyvinylidene difluoride (PVDF) membranes. Antibodies used were ActRIIB (Abcam ab76940; Cambridge, MA, USA), at a concentration of 1:500, and secondary enhanced chemilumescent (ECL)-anti-mouse IgG, 1:5,000 (GE Healthcare NA931V; Pittsburgh, PA, USA). Following visualization with the Amersham ECL Prime Western Blotting Detection Reagent (GE Healthcare), quantification was performed using the NIH ImageJ program.

Myostatin (GDF-8) levels from the tissue lysates were determined using an immunoaffinity liquid chromatography-mass spectrometry (LC-MS) workflow. Tissue lysates were prepared by homogenization of each muscle sample using tissue protein extraction reagent (TPER, Pierce cat 78510) (ThermoFisher Scientific). Homogenate was centrifuged and 20 μL of the supernatant was used for myostatin analysis. Quantification of myostatin was performed against recombinantly produced myostatin protein (R&D Systems, Inc. Minneapolis, MN, USA; catalog number 788-G8). Recombinant protein standards were prepared in 1 % bovine serum albumin (BSA) in phosphate buffered saline containing 0.05 % Tween-20 (PBST). A seven-point standard curve was prepared from 0.1 to 12.8 ng/mL by serial dilution. Tissue levels were back calculated against this standard curve and normalized using tissue weight; levels are reported in pg of myostatin per mg of tissue. Samples were prepared by first adding 200 μL of 0.1 M Glycine buffer (pH 2.5) to 20 μL of sample and incubating for 1 h at room temperature. After 1 h, 50 μL of 1 M tris buffer (pH 8) was added to each sample, followed by 1 μg of biotin conjugated anti-GDF-8 antibody and incubated overnight at 4 °C. After incubation, streptavidin magnetic beads were used to isolate the myostatin/antibody complex. The magnetic beads were washed twice with PBST, followed by one PBS wash. Myostatin was then separated from the antibody and beads using 140 μL of 30 mM hydrochloric acid prepared in water. The acid eluate was then neutralized using 30 μL of 1 M tris buffer (pH 8). All samples were subsequently reduced using dithiothreitol (DTT), cysteines were alkylated with iodoacetamide, and the protein was digested using trypsin. Quantification occurred by nano liquid chromatography-mass spectrometry and liquid chromatography (LC-MS-MS) using an API 5500Q mass spectrometer against the tryptic peptide IPAMVVDR.

#### Satellite cell quantitation

For satellite cell quantification of muscle, *Tabitha*, *Hagatha*, *Endora*, and *Esmerelda* were anesthetized at 37 months of age and samples of the CS and VL were removed surgically and processed as described above. Muscle cryosections (10 μm) were immunostained using antibodies to Pax7 (Developmental Studies Hybridoma Bank (DSHB), Iowa City, IA; 1:10) followed by goat anti-mouse secondary antibody (Alexa Fluor 488, Invitrogen A11001; 1:500) and an antibody to laminin (2E8, DSHB, 1:100), and then goat anti-rabbit secondary antibody (Alexa Fluor 594, Invitrogen, A11012; 1:500). All nuclei were labeled with 4′,6-diamidino-2-phenylindole (DAPI) in mounting medium (P36930, Invitrogen). Image capture was performed using an upright microscope for bright field, differential interference contrast (DIC), and epifluorescence with an ApoTome structured illumination digital imaging system (Carl Zeiss Meditec, Inc, Dublin, CA). The image stacks consisted of six optical sections with 1.4 mm Z-steps. Nuclei staining positive for DAPI but outside laminin extracellular membranes were considered interstitial cells, while those inside laminin were considered myonuclei. Satellite cells were identified as Paired Box 7 (Pax7)+ nuclei within laminin staining as shown in Additional file 1: Figure S1 and as previously described [53].

### Statistical analysis

The statistical package, R version 3.0.1 [54], was used for all statistical analyses. Analysis of variance (ANOVA) tests were used to compare results from individual dystrophic groups and controls. From the ANOVA tests, we examined whether at least one group was significantly different from the others. Once the null hypothesis was rejected in the ANOVA, we performed a post hoc Tukey’s honest significant differences (HSD) test to identify differences between a pair of groups. The model can be written as *y*_*ij*_ *= μ*_*i +*_ Ε_*ij*_, where the subscript *i* represents the group (1 = *GRippets*; 2 = GRMD dogs; 3 = non-dystrophic controls), the subscript *j* represents the *j*th subject, the *y*_*ij*_ is the response variable of the *j*th subject of the *i*th group, the *μ*_*i*_ is the mean of the *i*th group, and the error term Ε_*ij*_ is assumed to have a standard normal distribution. This model was repeated to assess messenger ribonucleic acid (mRNA) and protein data, plus each MRI marker, texture feature, functional measurement, and histopathological lesion of each muscle. We computed Pearson correlation coefficients between MRI, histopathological, and mRNA/protein data. To account for multiple comparisons, we applied the false discovery rate (FDR) method to calculate the corrected *p* values [55]. A value of *p* < 0.05 was considered significant. Trends were cited if *p* < 0.2.

## Results

**Fig. 1:**
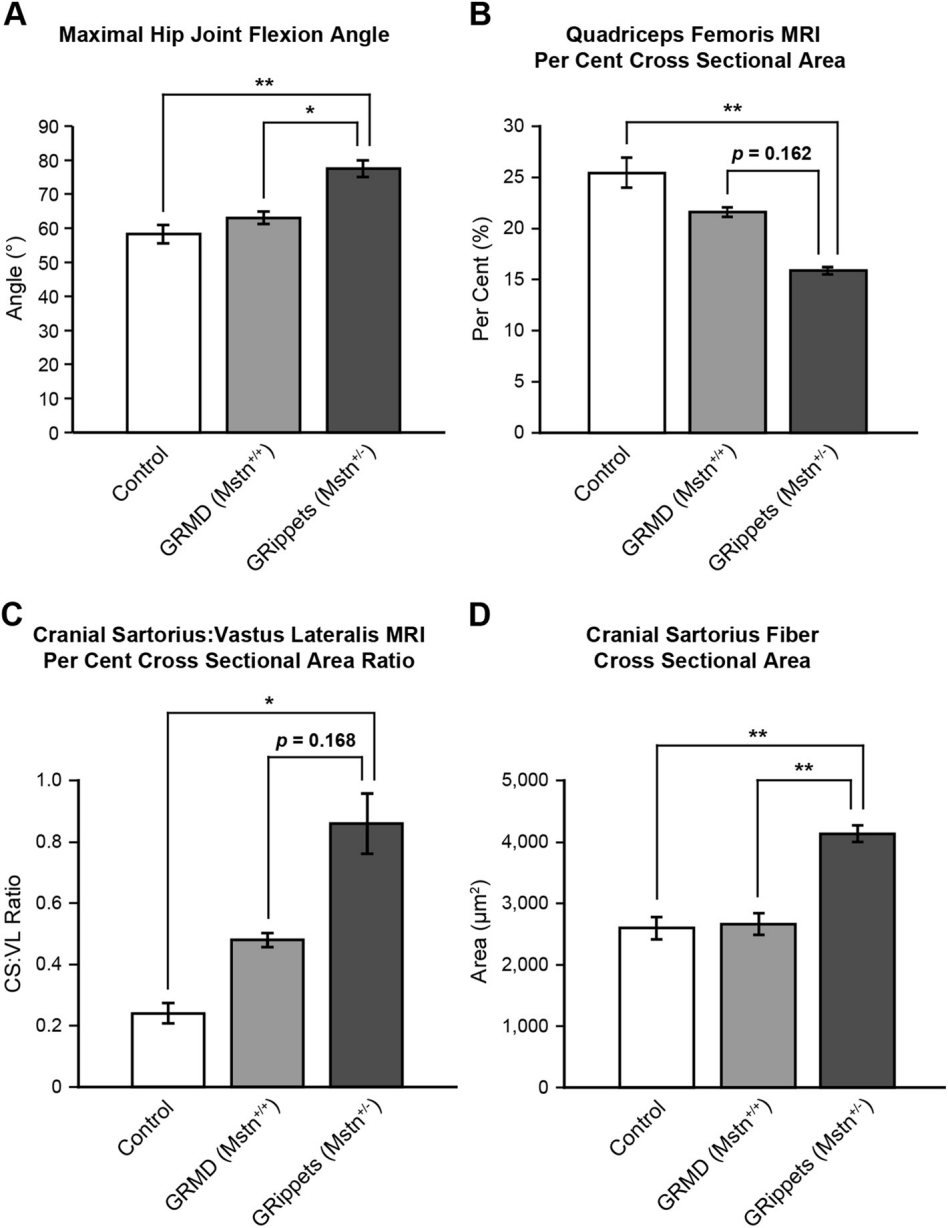
Histograms demonstrating differences among control, GRMD, and *GRippet* dogs. **a** Maximal hip joint flexion angle. **b** Quadriceps femoris MRI percent cross-sectional area. **c** Cranial sartorius to vastus lateralis MRI percent cross-sectional area ratio. **d** Cranial sartorius fiber cross-sectional area. For each of these phenotypic measures, differences between control and dystrophic dogs are more pronounced in the *GRippets* versus GRMD dogs

Key molecular and phenotypic features of the dogs of this study are discussed below. Data are expressed as mean ± SD. Because of the small group sizes and stringency of the FDR statistical method, many of the differences between the two dystrophic groups did not reach significance. Significant (*p* < 0.05) findings and those that trended towards significance (*p* < 0.2) between GRMD and *GRippet* dogs are summarized in Table 2; some are illustrated in Fig. 1.

**Table 2:** Differences between GRMD (*Mstn* ^*+/+*^) and *GRippet* (*Mstn* ^*+/−*^) dogs (significant (*p* < 0.05) and trending (*p* < 0.2))

Test	*GRippets* (*Mstn* ^*+/−*^) (mean ± SD)	GRMD (*Mstn* ^*+/+*^) (mean ± SD)	*p* value
Joint angles			
Maximal hip joint flexion angle (^o^)	77.5 ± 4.93	63.0 ± 3.61	0.05
Maximal tarsal joint flexion angle (^o^)	53.5 ± 7.85	35.0 ± 7.0	0.05
Maximal stifle joint flexion angle (^o^)	42.0 ± 5.35	34.0 ± 3.61	0.173
Tarsal range of motion (^o^)	104 ± 8.73	123 ± 4.04	0.052
Magnetic resonance imaging			
Rectus femoris percent cross-sectional area	2.70 ± 0.76	4.29 ± 0.30	0.168
Quadriceps femoris percent cross-sectional area	15.9 ± 0.61	21.6 ± 0.94	0.162
CS/VL ratio (volume and percent cross-sectional area)	0.86 ± 0.19	0.48 ± 0.04	0.168
Molecular/histopathology			
Lateral head gastrocnemius myostatin mRNA fold change	1.35 ± 0.682	0.565 ± 0.454	0.192
Overall average degenerating fibers (%)	2.58 ± 0.36	3.97 ± 1.21	0.082
Cranial sartorius fiber cross-sectional area (mm^2^)	4,133 ± 233	2,661 ± 316	0.01
Cranial sartorius centrally nucleated fibers (%)	20.7 ± 3.82	11.4 ± 5.23	0.132

### Posture, gait, and outcome

**Fig. 2:**
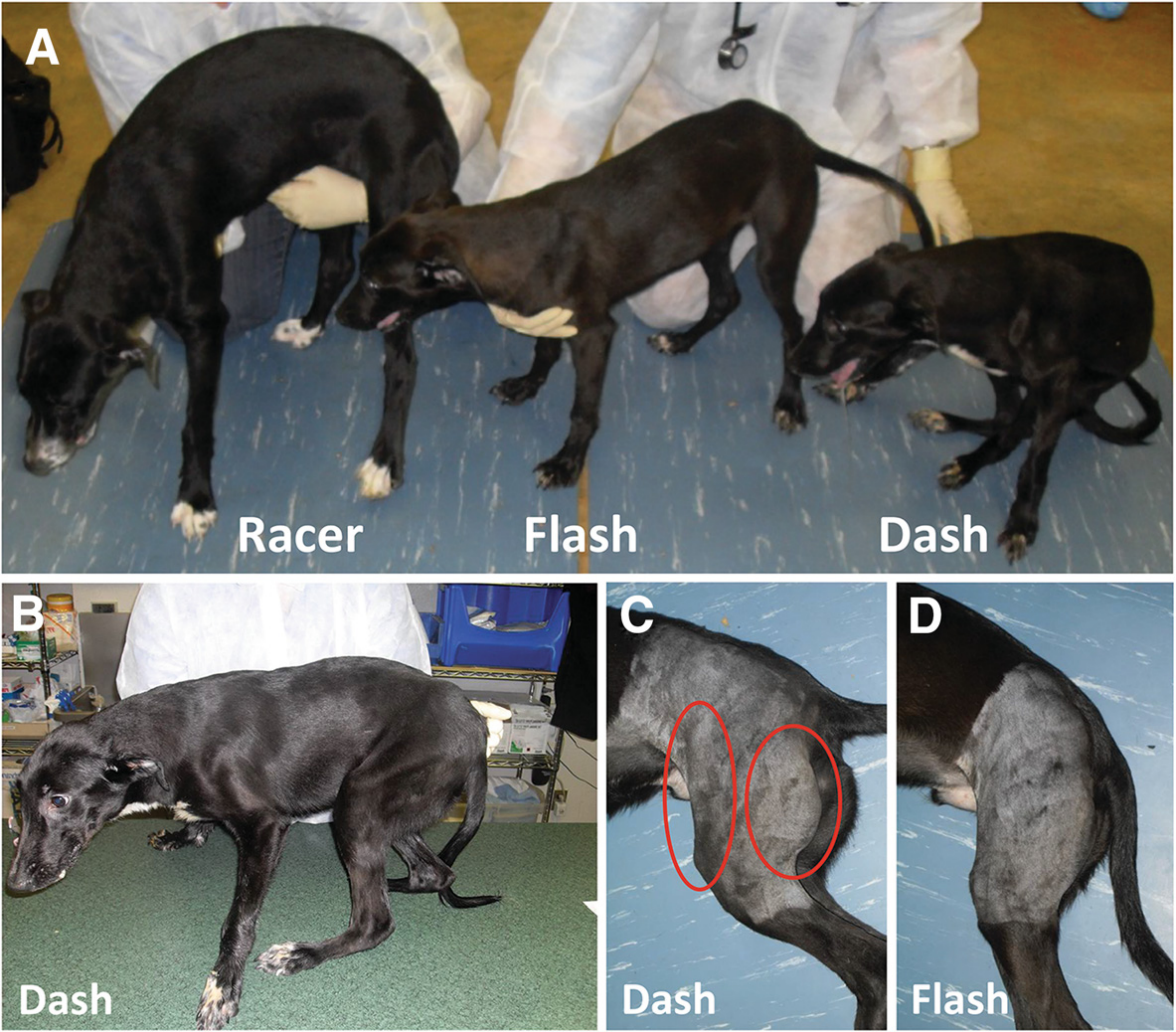
Comparative posture and muscle mass of dogs from the first litter. **a** Left to right, *Racer* (muscular dystrophy normal; *Mstn* ^*+/+*^), *Flash* (GRMD; *Mstn* ^*+/+*^), and *Dash* (*GRippet*; *Mstn* ^*+/−*^), illustrating stunting in *Flash* and *Dash* and dramatic postural changes in *Dash*. **b** Dash (*GRippet*; *Mstn* ^*+/−*^): note the forward shift and plantigrade positioning of the pelvic limbs. **c**, **d** The CS and hamstring muscles (*circles*) in *Dash* (**c**) are relatively hypertrophied compared to *Flash* (**d**)

**Fig. 3:**
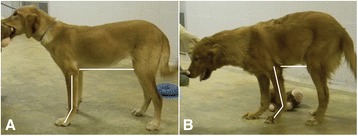
Comparative posture of dystrophic dogs from the second litter. Note the comparative posture of the dystrophic GRMD (*Hagatha*) (**a**) and *GRippet* (*Derwood*) (**b**) dogs. *Derwood*’s carpal joints are hyperextended, resulting in a more plantigrade stance in the thoracic limbs (*lines* are drawn to delineate the carpal joint in each dog). *Hagatha* has a normal upright posture, while *Derwood*’s lumbar spine is kyphotic and his thoracic and pelvic limbs are shifted under the trunk (represented by the shorter line extending from the elbow to the stifle)

**Fig. 4:**
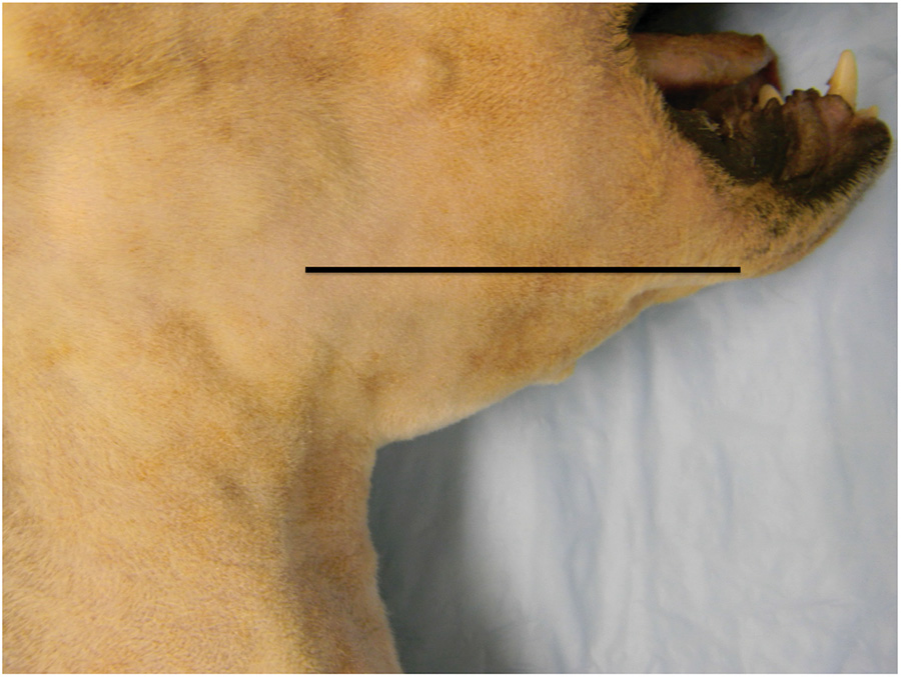
Glossal hypertrophy in dystrophic *GRippet* dog. Lateral view of the pharyngeal area of *GRippet* dog, *Derwood*, illustrating glossal hypertrophy. The tongue extends well ventral to the line demarcating the mandible and ventral aspect of the oral cavity [76]

Objective measures of gait, such as accelerometry and video gait analysis, were not evaluated in these dogs. However, key postural changes and quantitative joint angle measurements (below) were compared between the two dystrophic groups. Overall, the dystrophic dogs had a more plantigrade posture and gait, with the limbs shifted forward under the torso. A plantigrade posture results from the carpi being hyperextended (dorsal flexion) and the tarsi being hyperflexed (palmar flexion). Postural abnormalities were more pronounced in the *GRippets*, equating to a more severe GRMD phenotype. These changes varied between the two litters. *Dash* (*GRippet*) from the first litter had a pronounced plantigrade stance, and his pelvic limbs were shifted forward (Fig. 2). He was no longer ambulatory at 9 months of age and was euthanized. Similar but milder postural changes were seen in the three *GRippets* of the second litter (*Derwood*, *Abner*, and *Tabitha*) (Fig. 3). While these dogs retained the ability to walk, they developed glossal hypertrophy, which interfered with their ability to eat (Fig. 4). Resulting weight loss necessitated euthanasia of *Abner* and *Derwood* at 9 and 11 months, respectively. The final *GRippet*, *Tabitha*, remained ambulatory but had pronounced postural abnormalities and was euthanized at 41 months.

### Joint angles

Compared to control dogs, joint angles of GRMD dogs were similar, whereas those of *GRippets* differed. The *GRippet* hip angle with maximal flexion was larger (more restricted) (77.5° ± 4.93), and hip ROM was smaller (56.5 ± 12.5) versus controls (58.3° ± 5.51, *p* < 0.01 for flexion; 87.7 ± 15.9, *p* < 0.05 for ROM) (Fig. 1a). An unexpected and important finding was that *GRippets* had larger (more restricted) maximal TTJ (53.5° ± 7.85), stifle (42.0 ± 5.35), and hip (77.5° ± 4.93) (Fig. 1a) flexion angles compared to GRMD dogs (35.0 ± 7.00, 34.0 ± 3.61, and 63.0 ± 3.61, respectively) (*p* < 0.05 for TTJ and hip; *p*  = 0.173 for stifle). The TTJ ROM motion trended towards being significantly decreased in the *GRippets* (*p* = 0.052) (Table 3).

**Table 3:** Joint angles in non-dystrophic control, GRMD (*Mstn* ^*+/+*^), and *GRippet* (*Mstn* ^*+/−*^) dogs

Genotype	Tarsal joint (mean ± SD)				Stifle joint (mean ± SD)			Hip joint (mean ± SD)		
	Original	Flexion	Extension	ROM	Flexion	Extension	ROM	Flexion	Extension	ROM
Controls	150 ± 11.1	45.0 ± 8.0	153 ± 6.43	108 ± 11.0	39.3 ± 6.03	146 ± 14.0	107 ± 11.4	58.3 ± 5.51^b**^	146 ± 10.4	87.7 ± 15.9^b*^
GRMD (*Mstn* ^*+/+*^ *)*	153 ± 12.0	35.0 ± 7.0^b*^	158 ± 11.0	123 ± 4.04	34.0 ± 3.61	138 ± 5.0	104 ± 7.94	63.0 ± 3.61^b*^	140 ± 10.8	76.7 ± 13.3
*GRippet* (*Mstn* ^*+/−*^ *)*	147 ± 19.4	53.5 ± 7.85^a*^	157 ± 12.5	104 ± 8.73	42.0 ± 5.35	140 ± 5.26	97.5 ± 6.56	77.5 ± 4.93^a*^	134 ± 13.3	56.5 ± 12.5

*ROM* range of motion

^a^Significantly different (*p < 0.05) from GRMD dogs

^b^Significantly different (*p < 0.05; **p < 0.01) from *GRippet*s

### Force and ECD

As with the joint angle data, *GRippet* body-mass-corrected force values (N/kg) varied markedly between the two litters. *Dash* from the first litter had dramatically higher TTJ tetanic flexion force (12.5) compared to the three GRMD dogs from the second litter (6.63 ± 1.59). A comparable but reversed differential was seen for extensor force, with *Dash* having a value of 11.1 versus 28.8 ± 2.75 for the other three *GRippets*. Flexion force values for *GRippet* (0.70 ± 0.41) and GRMD dogs (0.59 ± 0.32) were each lower than the controls (1.54 ± 0.04) (*p* < 0.05 for both). The GRMD (1.93 ± 0.41) and *GRippet* (1.92 ± 0.64) dogs also had lower extension force compared to controls (3.16 ± 0.48) (*p* < 0.05 for *GRippets* and *p* = 0.062 for GRMD). Values for ECD after either 10 or 30 contractions were higher in each dystrophic group versus controls (*p* < 0.05 for all). Neither force nor ECD values differed significantly between the two dystrophic groups (Table 4).

**Table 4:** Body-mass-corrected tetanic force (N/Kg) and ECD measurements in non-dystrophic control, GRMD (*Mstn* ^*+/+*^), and *GRippet* (*Mstn* ^*+/−*^) dogs

	Flexion (mean ± SD)	Extension (mean ± SD)	ECD (10) (mean ± SD)	ECD (30) (mean ± SD)
Controls	1.54 ± 0.04^a*,b*^	3.16 ± 0.48^b*^	9.38 ± 6.18^a*,b*^	22.1 ± 11.6^a*,b*^
GRMD (Mstn^+/+^)	0.59 ± 0.32	1.93 ± 0.41	38.6 ± 14.8	64.2 ± 7.56
*GRippet* (Mstn^+/−^)	0.70 ± 0.41	1.92 ± 0.64	33.2 ± 2.77	61.7 ± 15.7

*ECD* percent eccentric contraction decrement after 10 and 30 tetanic flexion contractions

^a^Significantly different (*p < 0.05) from GRMD dogs

^b^Significantly different (*p < 0.05) from *GRippet*s

### Cranial sartorius circumference

Cranial sartorius circumference corrected for body mass (mm/kg) trended towards being higher in the *GRippets* (5.05 ± 1.72; *p* = 0.053) but not GRMD dogs (3.39 ± 0.52, *p* = 0.617) when compared to controls (2.46 ± 0.25). Values for the two dystrophic groups did not differ (*p* = 0.219).

### MRI

#### Volumetric findings

**Fig. 5:**
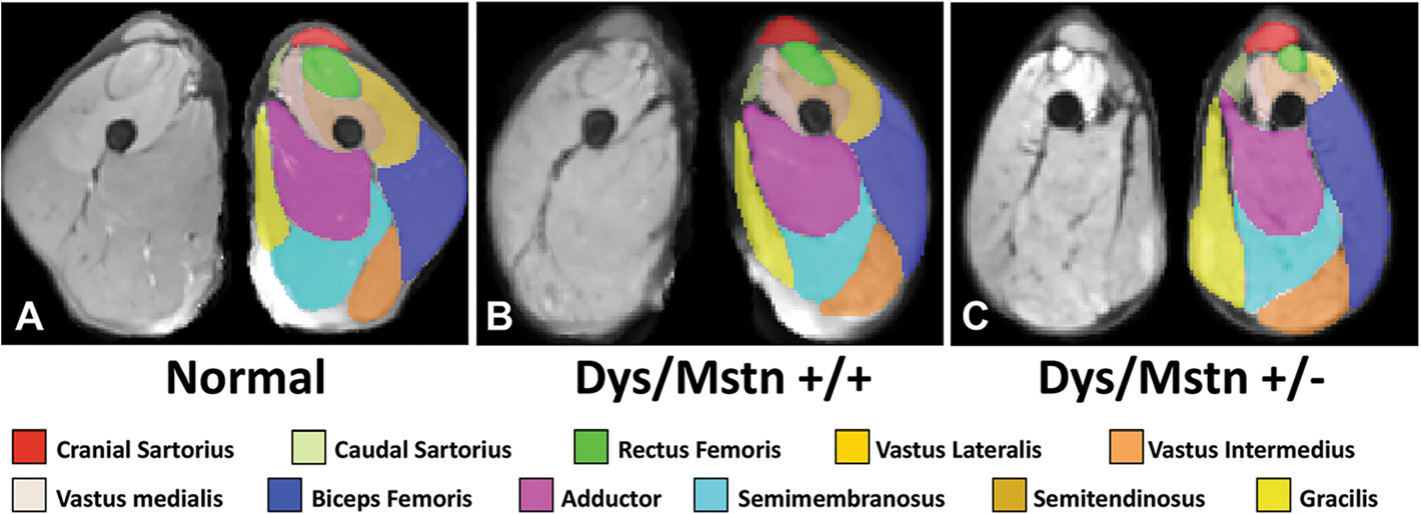
Averaged MRI segmentation of dogs from the three groups. Averaged T2-FS MRI images of pelvic limb muscles in the transverse plane at the level of the midthigh are shown in non-dystrophic control (**a**) dystrophic GRMD *Mstn* ^*+/+*^ (**b**) and *GRippet Mstn* ^*+/−*^ (**c**) dogs. Note the proportional enlargement of the sartorius and hamstring muscles and the associated atrophy/hypoplasia of the quadriceps femoris of the dystrophic GRMD *Mstn* ^*+/+*^ dogs, relative to the non-dystrophic control dogs, and the even more dramatic differential size of these muscles in the *GRippet Mstn* ^*+/−*^ dogs (also see quantitative measurements in Additional file 2: Table S1)

Effects of muscle atrophy or hypertrophy in dystrophic dogs were demonstrated by MRI, with parallel changes being seen with both absolute (mm^3^) and femur length-corrected (mm^3^/mm) volumes and the percent contribution that each muscle made to the overall CSA (Fig. 5 and Additional file 1: Figure S1; Additional file 2: Table S1).

Muscle volumes varied considerably among the dogs and were not clearly associated with *Mstn* gene status. As an example, overall mid-femur length-corrected muscle volume in the transverse plane ranged from 114 in *Hagatha* (GRMD) to 191 in *Endora* (*GRMD* carrier/*Mstn*^*+/+*^). *Racer*, the only dog that was normal for both genetic traits, had an intermediate value of 164. *Esmeralda* (*GRMD* normal/*Mstn*^*+/–*^) had a higher value (173), potentially in keeping with a hypertrophic effect of myostatin loss, but *Endora*’s value was even higher (191).

A differential effect of the dystrophy trait on individual muscles was demonstrated by assessing volumes of the quadriceps femoris (QF) and the combined cranial and caudal sartorius (total sartorius) muscles. The corrected QF volume ranged from 19.2 in *Dash* (*GRippet*) to 47.0 for *Racer*, while an opposite pattern was seen for the total sartorius, with values ranging from 4.3 for *Racer* to 13.0 for *Dash*.

#### *GRippet* and GRMD dogs versus non-dystrophic controls

When data from individual dystrophic groups were compared, controls had greater absolute (29,631 ± 274), and femur length-corrected (178 ± 13.4) volumes than either GRMD (19,682 ± 1255, absolute; 125 ± 8.87, corrected) or *GRippet* (21,791 ± 1409, absolute; 137 ± 7.76, corrected) dystrophic dogs (*p* < 0.01 for all). Differential effects on individual muscles paralleled those of the individual dogs. Control QF volumes were larger than those of either dystrophic group (*p* < 0.01 for both). Total sartorius volumes of the controls were smaller than those for either *GRippet* or GRMD dogs, with the *GRippet* value trending towards significance (*p* = 0.159 for absolute and 0.112 for corrected). In comparing either absolute or corrected volumes of the other muscles of the two dystrophic groups separately to controls, only the biceps femoris differed, being significantly smaller in both the GRMD and *GRippet* dogs (*p* < 0.01 for most).

With regard to CSA, the QF accounted for 25.4 ± 2.99 % in the controls, versus 21.6 ± 0.9 % in the GRMD dogs and 15.9 ± 0.61 % in the *GRippets* (Fig. 1b). Values for the *GRippets* differed significantly from controls (*p* < 0.01) but those for the GRMD dogs did not (*p* = 0.864). The CS/VL ratio showed a clear progression from the controls (0.24 ± 0.06), to the GRMD dogs (0.48 ± 0.04), to the *GRippets* (0.86 ± 0.19) (Fig. 1c), indicating that reduction in myostatin accentuated unequal muscle growth in dystrophic dogs. Differences for *GRippets* versus controls were significant (*p* < 0.05), but those for GRMD dogs were not (*p* = 0.864).

#### *GRippet* versus GRMD dogs

While *GRippet* absolute and femur length-corrected total muscle volumes were similar to those of the GRMD dogs, the pattern of differential muscle involvement between dystrophic and control dogs was exaggerated in the *GRippets*. For almost all muscles, the degree of atrophy or hypertrophy in GRMD dogs was more pronounced in the *GRippets* (Additional file 1: Figure S1). The differential effects on the quadriceps and sartorius muscles were highlighted by the CS/VL ratio, which trended towards being higher in the *GRippets* (*p* = 0.168).

Individual muscle volume on MRI and joint angles of the 10 dogs were correlated to clarify whether imbalanced muscle volume/strength contributed to postural changes. We expected that CS muscle volume would correlate negatively with TTJ angle measured by our *original* method [38] but neither the muscle volumes nor the percent CSA values correlated. This outcome may have been influenced by the age of the dogs. With our prior studies, the correlation was noted at 6 months of age; correlations have not been demonstrated at other ages. On the other hand, CS and total sartorius absolute volumes and percent CSA correlated positively with maximal hip flexion angle (*r* = 0.671 to 0.756; *p* < 0.05 for all but CS absolute volume for which *p* = 0.063). There was also a strong negative correlation between absolute and femur length-corrected QF volume (*r* = −0.829 and 0.809) and percent CSA (*r* = −0.935) versus maximal hip flexion (*p* < 0.05 for absolute and corrected volumes and 0.01 for percent CSA). These correlations could be in keeping with biomechanical relationships or represent independent variables tracking with the disease phenotype.

#### T2 mapping values and texture analysis

The overall T2 mapping value for the non-dystrophic control dogs (38.2 ± 5.00) was lower than that for the *GRippets* (52.3 ± 3.47) and GRMD dogs (51.9 ± 5.09) (*p* < 0.05 for both). T2 mapping values of individual muscles, with the exception of the sartorius heads, were also higher, although differences generally only trended towards significance. Values for texture analysis features did not differ significantly among the three groups (data not shown) (Additional file 3: Table S2).

### Pathologic and molecular findings

#### Histopathologic changes (degenerating and regenerating fibers; centrally nucleated fibers)

Averaged scores for degenerating fibers and CNF were higher in *GRippet* and GRMD dogs versus controls (*p* < 0.01 for all but CNF in GRMD which was <0.05). In contrast, the difference for regenerating fiber scores in the two dystrophic groups only trended towards significance in GRMD dogs (*p* = 0.101) and was not significant in the *GRippets* (*p* = 0.523). Most scores for degenerating and regenerating fibers for individual muscles of separate dystrophic groups did not differ from control individual muscles. The one exception was the presence of greater numbers of degenerating fibers in the LDE of the GRMD dogs (*p* < 0.05). In contrast, CNF were increased in both dystrophic groups compared to controls in all muscles (*p* < 0.05 for most). Importantly, histopathologic lesions in *GRippet* and GRMD muscle did not differ. Specifically, there were no statistically significant differences in degenerating, regenerating, or CNF in *GRippets* compared to their GRMD littermates (Additional file 4: Table S3).

#### Pax7-positive nuclei

To investigate the potential that modulation of myostatin in muscular dystrophy might lead to exhaustion of the satellite cell pool, we quantified Pax7-positive nuclei in two dystrophic dogs, including one *GRippet* (*Tabitha*) and another *Mstn*^*+/+*^ GRMD dog (*Hagatha*), as well as two GRMD carriers that differed on myostatin status (*Endora* (*Mstn*^*+/+*^) and *Esmerelda* (*Mstn*^*+/−*^)). All dogs were adults, 37 months of age. There were no statistically significant differences in satellite cell numbers between muscles from dogs of different myostatin genotypes. However, somewhat unexpectedly, Pax7-positive cells accounted for a higher percentage of myonuclei in the two dystrophic dogs (4.3–5.7 %) versus the two GRMD carriers (0.6–2.8 %) for the four muscles studied (Table 5; Additional file 5: Figure S2).

**Table 5:** Pax7-positive cells in dystrophic and carrier dogs

Dog/muscle	Genotype	Pax7+	Myonuclei	Percentage
*Endora*/CS	GRMD carrier/*Mstn* ^*+/+*^	12	918	1.3
*Endora*/VL	GRMD carrier/*Mstn* ^*+/+*^	7	1151	0.6
*Esmeralda*/CS	GRMD carrier/*Mstn* ^*+/−*^	8	983	0.8
*Esmeralda*/VL	GRMD carrier/*Mstn* ^*+/−*^	33	1173	2.8
*Hagatha*/CS	GRMD/*Mstn* ^*+/+*^	68	1193	5.7
*Hagatha*/VL	GRMD/*Mstn* ^*+/+*^	63	1462	4.3
*Tabitha*/CS	*GRippet*/*Mstn* ^*+/−*^	89	1651	5.4
*Tabitha*/VL	*GRippet*/*Mstn* ^*+/−*^	95	1681	5.7

#### CSA of myofibers

Consistent with the increase in muscle fiber size in the genetic absence or postnatal blockade of myostatin [6, 14]*,* the CSA for the *GRippet* CS muscle (4133 ± 233) was larger than that of their GRMD littermates (2661 ± 316) (*p* < 0.01). In comparing the CSA of the CS fibers to controls (2,598 ± 346), *GRippets* had larger fibers (*p* < 0.01), while those of the GRMD dogs did not differ (*p* = 0.964). These findings of muscle fiber hypertrophy were not seen in other muscles. In fact, in the VL, the CSA was smaller in both dystrophic groups (*GRippets,* 1560 ± 155, *p* < 0.01; GRMD*,* 1850 ± 610; *p* < 0.05), compared to the controls (3,196 ± 326) (Fig. 1d) (Additional file 4: Table S3).

#### Hydroxyproline (HP) protein

Fibrosis is a major complicating factor of chronic myopathy. Studies have shown that myostatin stimulates muscle fibroblasts and that fibrosis is reduced in the absence of myostatin [51]. Hydroxyproline content (μg HP/mg of total muscle protein) is a biomarker of muscle fibrosis and collagen content. Thus, we were interested in the relative HP content of the dystrophic and control groups. Overall HP content of GRMD dogs (26.5 ± 9.30) was higher than controls (9.53 ± 2.56) (*p* < 0.05), while that of the *GRippet* dogs (22.9 ± 4.79) trended towards being higher (*p* = 0.053). Although HP content for individual muscles from the dystrophic groups did not differ significantly when compared to controls, there were trends towards greater content in the *GRippet* VL (*p* = 0.074) and GRMD VL, CS, and LHG (all *p* < 0.2). Interestingly, the differential between the two dystrophic groups varied markedly among muscles, with *GRippet* values being lower in the CS and LHG but higher in the VL and LDE. None of these differences were significant (Additional file 4: Table S3).

#### Myostatin mRNA and protein expression

**Fig. 6:**
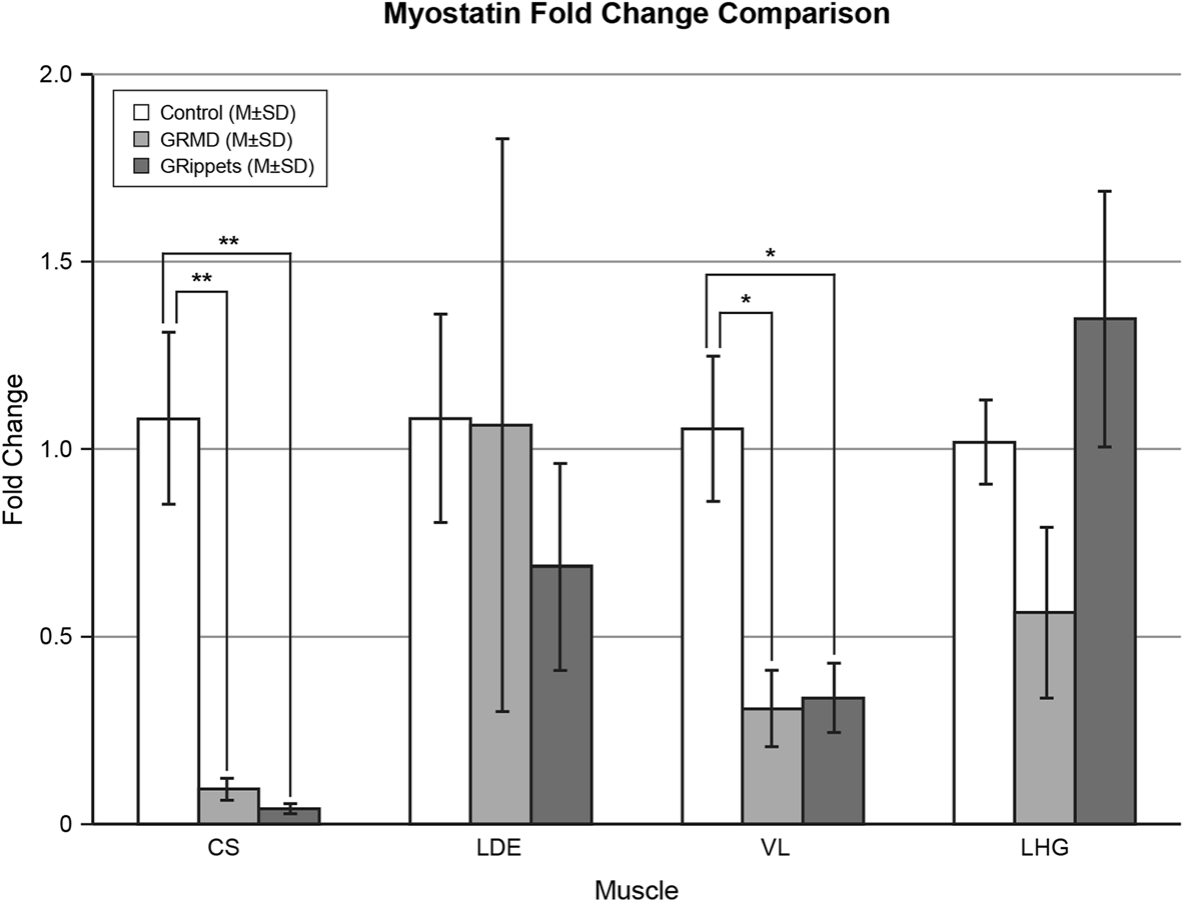
Myostatin mRNA levels in control, GRMD, and *GRippet* dogs. Values were significantly reduced in the CS of GRMD and *GRippet* dogs, compared to controls (*p* < 0.01 for both). A less pronounced reduction was seen in the VL of GRMD and *GRippet* dogs (*p* < 0.05 for both) versus controls. Values for the LDE and LHG did not differ among the three groups. No differences were seen between GRMD and *GRippet* dogs. All measurements are given in relation to HPRT values, which were normalized to 1

Myostatin mRNA data from the CS and VL of these same dogs have been reported previously [37]. Levels in the CS of both dystrophic groups were significantly lower than controls, while those in the VL were lower but not significantly different. Gene expression in these muscles plus the LDE and LHG was assessed for this current study. Levels averaged for all four muscles were reduced to a comparable degree in *GRippet* and GRMD dogs at 8–9 months when compared to controls but differences were not significant. In assessing individual muscles, levels were reduced in the CS of GRMD (0.093 ± 0.059) and *GRippet* (0.041 ± 0.026) dogs, compared to controls (1.08 ± 0.458) (*p* < 0.01 for both) (Fig. 6). A less pronounced reduction was seen in the VL of GRMD (0.306 ± 0.20) and *GRippet* (0.335 ± 0.185) dogs versus controls (1.05 ± 0.388) (*p* < 0.05 for both) (Additional file 4: Table S3).

Similar to mRNA findings, average myostatin protein levels (pg/mg) for the three muscles assessed were lower in *GRippet* (2.94 ± 1.48) and GRMD (4.30 ± 0.98) dogs compared to controls (7.63 ± 3.51) but differences were not significant, nor did values differ between dystrophic groups (*p* = 0.770). Levels were significantly lower in the dystrophic versus control CS regardless of *Mstn* genotype (0.92 ± 0.73 in the *GRippets* and 1.63 ± 0.39 in GRMD) vs. 7.79 ± 2.18 in the two non-dystrophic dogs) (*p* < 0.05 for both). This is in keeping with the dramatic hypertrophy seen in the CS in even *Mstn*^*+/+*^ GRMD dogs [44, 56] and suggests that the hypertrophy may be more pronounced when myostatin is further down regulated. Consistent with this observation, *Dash,* the *GRippet* with the most pronounced postural changes and CS muscle hypertrophy, also had the lowest myostatin protein levels (0.21) by a wide margin. Values for the LDE and LGH did not differ between the dystrophic and control dogs.

Myostatin mRNA fold change and protein levels were correlated with MRI volumes and histopathologic changes among the dogs (all 10 for mRNA and 8 for protein) to help establish potential cause and effect links. Notably, there was a significant negative correlation between CS myostatin mRNA fold change (*r* = −0.7297) and protein (*r* = −0.7649) levels and CS percent CSA on MRI (both *p* < 0.05), suggesting myostatin down regulation may contribute to CS hypertrophy. A similar but positive correlation was seen between *Mstn* mRNA levels and VL volume corrected for femur length (*r* = 0.680; *p* < 0.05), providing a further potential link between down regulation of myostatin and VL atrophy.

With regard to histopathological findings, both myostatin mRNA and protein levels in the CS correlated negatively with the levels of degenerating fibers and CNF (*r* = −0.8178, *p* < 0.01 for mRNA; *r* = −0.7617, *p* < 0.05 for protein). A less pronounced effect was seen in the VL, with mRNA levels correlating negatively with degenerating fibers (*r* = −0.6433, *p* < 0.05) and a trend occurring for CNF (*r* = −0.5538, *p* = 0.097). These data indicate that the exaggerated lowering of myostatin could place muscle fibers at increased risk for injury (degenerating fibers) and subsequent regeneration (CNF).

#### Activin receptor type IIB (ActRIIB) 

**Fig. 7:**
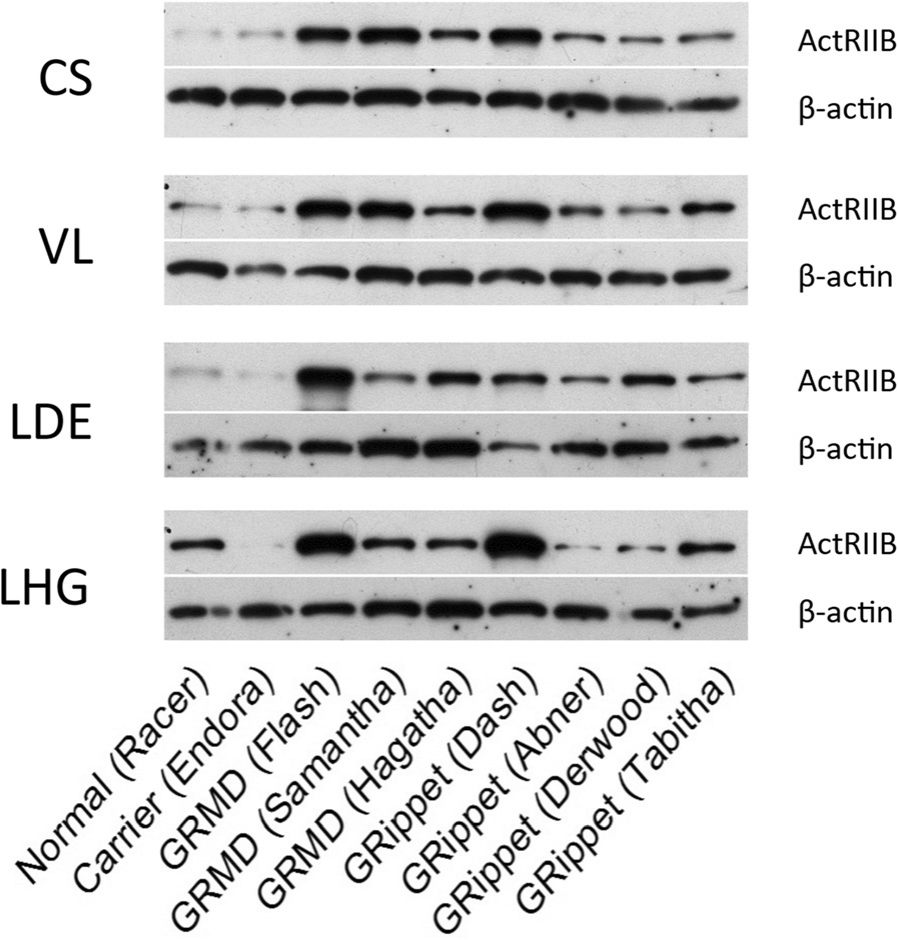
Western blots of ActRIIB levels normalized to β-actin. Blots from muscles of the two myostatin wild-type (*Racer* and *Endora*), three GRMD (*Flash*, *Samantha*, and *Hagatha*), and four *GRippet* (*Dash*, *Abner*, *Derwood*, and *Tabitha*) dogs are shown. Levels are upregulated in the dystrophic dogs compared to controls, but there is no consistent difference among individual muscles or between the *GRMD* and *GRippet* dogs. Levels are expressed in arbitrary units

Expression of the putative receptor for myostatin, ActRIIB, varies among muscles in mice. For example, the fast-twitch EDL has higher levels than the slow twitch soleus [20]. Thus, we were interested in whether the relative expression of ActRIIB protein among muscles of dogs in this study correlated with differential muscle size. ActRIIB protein expression normalized to beta-actin varied markedly among the four muscles. For *Racer*, the only completely normal dog, levels in the CS (0.077 arbitrary units) were considerably lower than those in the VL (0.299), LDE (0.408), and LHG (0.259). In contrast, for *Endora* (*GRMD* carrier, *Mstn*^*+/+*^), the CS ActRIIB level (0.190) was higher than that of either the LDE (0.090) or LHG (0.011) but lower than the VL (0.391). Thus, based on these limited data from the two non-dystrophic, *Mstn*^*+/+*^ dogs, there was no clear pattern of differential ActRIIB expression among the four muscles to account for the pattern of muscle atrophy/hypertrophy protein (Additional file 4: Table S3 and Fig. 7).

ActRIIB levels have not been assessed previously in GRMD dogs. Interestingly, levels for all muscles of the combined dystrophic dogs (0.98 ± 0.42) were higher compared to average overall values for *Racer* and *Endora*, the two non-dystrophic dogs with wild-type myostatin (0.22 ± 0.06) (*p* < 0.01). Moreover, ActRIIB levels in individual muscles were higher in dystrophic dogs (CS, *p* < 0.01; VL and LDE, *p* < 0.05) in all but the LHG (*p* = 0.585). However, neither overall mean (*GRippet* 0.92 ± 0.52; GRMD 1.06 ± 0.32) (*p* = 0.903) nor individual muscle ActRIIB expression differed between the *Mstn*^*+/+*^ and *Mstn*^+/−^dystrophic groups.

The level of feedback between myostatin and ActRIIB, or other growth factors and receptors, could differ among muscles, leading to variable atrophy or hypertrophy. We correlated levels of ActRIIB protein with myostatin mRNA and protein to further establish a potential relationship. We were particularly interested in the CS muscle, given its marked hypertrophy. For all dogs taken together, levels of CS ActRIIB trended to correlate negatively with those of myostatin mRNA (*r* = −0.726; *p* = 0.108) and protein (*r* = −0.6739; *p* = 0.142). This would be in keeping with an expected physiologic relationship between the two, with ActRIIB levels increasing as myostatin levels decline. The degree of correlation between myostatin mRNA and ActRIIB levels was assessed in the dystrophic groups alone to gain further insight into potential disease associations. No correlations were seen.

## Discussion

Development of myostatin inhibitors for muscle-wasting disorders is based on the assumption that enhanced muscle regeneration will improve muscle function. Because mdx mice, which lack myostatin [13, 14] or in which myostatin is blocked [15] are less severely affected, we hypothesized that the relative genetic loss of myostatin would improve signs in dystrophic dogs. Interpretation of data in this study is compromised by the small group sizes and the inherent phenotypic variation seen in GRMD dogs. With that said, phenotypic features of GRMD dogs heterozygous for myostatin (*GRippets*) and their GRMD littermates were similar. Thus, we were unable to demonstrate a beneficial effect of reduced myostatin in GRMD dogs. In fact, when compared to their GRMD dystrophic littermates, *GRippets* actually had more severe postural changes, evidenced by an exaggerated plantigrade stance, shifting of the pelvic limbs forward, and more restricted hip joint flexion. Strikingly, the relative absence of myostatin in the *GRippets* exaggerated pre-existing trends for either muscle atrophy or hypertrophy on MRI, most notably in the quadriceps and sartorius muscles, as shown by the progressive increase of the CS to VL volume ratio of the non-dystrophic controls (0.24 ± 0.06), to the GRMD dogs (0.48 ± 0.04), to the *GRippets* (0.86 ± 0.10). This differential pattern of muscle atrophy/hypertrophy appeared to have functional consequences, in that CS and VL volumes correlated, in opposite directions, with maximal hip flexion angle.

We have previously demonstrated unequal disease effects on TTJ flexors and extensors in natural history and preclinical studies in GRMD dogs [36, 42] and shown that proportional weakness of TTJ extensors or increased strength of flexors exaggerates TTJ joint flexor contractures [38]. Moreover, the degree of CS hypertrophy in GRMD correlates negatively with the TTJ joint angle [44], suggesting that this muscle could play a role analogous to iliotibial band tightening in DMD [57]. Differential muscle involvement also plays a role in the DMD disease course. Unbalanced forces acting on a joint, due to either disproportionate muscle weakening or shortening, lead to abnormal positioning. Several studies have assessed the proportional strength of joint agonist and antagonist muscles in DMD, with most concluding that muscle imbalance contributes to contractures [58–62]. Those noting a relationship identified a strong negative correlation between extensor muscle weakness and flexor contracture severity in DMD. As opposing extensor muscles weakened, flexor contractures worsened. To our knowledge, such contractures and postural changes are not seen in *bully whippet* dogs *(Mstn*^*−/−*^*)* despite their gross muscle enlargement. Instead, a single *bully whippet* included in the original study was judged to run faster than myostatin wild-type dogs [12]. This suggests that contractures are only precipitated when myostatin down regulation is coupled with the dystrophic state.

Key questions arising from our study relate to (1) why relative loss of myostatin would cause differential muscle effects that exaggerate the GRMD phenotype and (2) whether this untoward effect could potentially extend to postnatal treatment of humans with muscle-wasting disorders. Treatments directed at inhibiting or blocking myostatin are driven by a belief that myostatin is innately limiting the muscle regenerative response. Importantly, in considering potential therapies for DMD, one must take into account homeostatic compensatory mechanisms that are already in play. In cases of muscle loss, the body should naturally suppress myostatin expression to promote muscle regeneration. Indeed, microarray gene expression profiling in DMD [63], the mdx mouse [64], and GRMD dog [56] has shown that myostatin is down regulated in dystrophic muscle independent of modulating treatments. Consistent with findings reported here, the degree of myostatin mRNA down regulation in GRMD dogs also has been shown previously to vary among muscles, with levels being dramatically reduced in the CS, a hip flexor prone to hypertrophy (44, 56), and less so or not at all in selected extensor muscles [56]. Not surprisingly, myostatin mRNA and protein levels in the CS of the *GRippet* dogs of our study were also markedly reduced when measured at 8–9 months of age. Interestingly, at this relatively late age, the *GRippet* levels were not significantly lower than those in their myostatin wild-type GRMD littermates. Had levels been measured earlier, particularly in advance of the rapid period of disease progression beginning at 3 months [36], we may have been able to distinguish a difference between GRMD and *GRippet* dogs. With this said, correlations between myostatin mRNA and protein levels with phenotypic features such as CS muscle volume on MRI and the degree of degenerating and regenerating fibers in both the CS and VL still suggested that the myostatin expression could contribute to variable muscle effects and the overall disease phenotype

The differential response of murine muscles to loss of myostatin is associated with varying levels of the myostatin receptor, ActRIIB [20]. In comparing ActRIIB levels among the three groups of dogs in our study, dystrophic dogs taken together had significantly higher levels than non-dystrophic controls, suggesting a feedback mechanism whereby ActRIIB levels increase secondary to lowering of myostatin. Similarly, when all dogs were assessed collectively, myostatin mRNA and protein levels tended to correlate negatively with ActRIIB expression in the CS muscle and, to a lesser extent, the VL. This would be in keeping with an expected physiologic relationship between the two, with ActRIIB levels increasing as myostatin levels decline. On the other hand, when the dystrophic groups were assessed alone, no correlation was seen. This likely is simply a function of small group sizes and the stringency of the FDR statistical method but could point to dysregulation in dystrophic muscle.

Myostatin favors differentiation towards slow versus fast glycolytic fibers, due apparently to positive and negative regulation of MEF2 and MyoD, respectively [65]. Consistent with this effect, cattle lacking *Mstn* [66] and *Mstn*-null mice [67] have increased numbers of fast glycolytic fibers. Therefore, in principle, fiber type distribution could influence effects of myostatin loss or blockade. Muscles with a predominantly fast glycolytic fiber type distribution could be more sensitive to the absence of myostatin and display greater hypertrophy. While most murine muscles respond similarly to genetic depletion of myostatin [5], the slow twitch soleus undergoes less pronounced hypertrophy than the fast-twitch EDL [20, 22, 24]. Based on the fast/slow fiber type distribution of canine muscles, the CS (49:51), VL (57:43), and LHG (50:50) are mixed muscles, while the LDE (71:29) is predominantly fast-twitch [68]. Independent of myostatin status, the GRMD LDE undergoes dramatic atrophy, the VL and LHG are moderately atrophied, and the CS is markedly hypertrophied [44, 56]. Therefore, fiber type distribution, alone, does not appear to be a major factor in GRMD muscle atrophy/hypertrophy.

With regard to the second question posed above on the potential for our findings to translate to humans, it is not clear whether deleterious effects of genetic loss of myostatin will extend to postnatal treatments. During development, myostatin down regulates myogenic regulators such as MyoD to inhibit proliferation and differentiation of myoblasts [5, 69]. Genetic loss of myostatin in the mouse accelerates both primary (embryo) and secondary (fetal) myogenesis, with an increase in myofiber numbers (hyperplasia) and size (hypertrophy) [70]. Myostatin-null mice have attained 87 % of their final adult number of myofibers by embryonic day 18.5 (near term) compared to only 73 % of wild-type mice [70]. Further postnatal muscle enlargement relies more on an increase in protein synthesis rather than incorporation of more nuclei [70]. Thus, one would logically suspect that effects of genetic versus postnatal loss or inhibition of myostatin would differ. However, the postural abnormalities seen in the *GRippet* dogs of our study did not become apparent until around 3 months of age. Accordingly, while the basis for differential muscle involvement may have been established in the embryo, phenotypic effects were not seen until after birth. Further, a previous study investigated the effect of postnatal stimulation of muscle growth in four GRMD dogs for 4 months beginning at 2.5 months of age. Results of this study were presented at the 16th International Congress of the World Muscle Society in 2011 [71] but have not been published. This study used treatment with anti-ActRIIB antibody, an inhibitor of multiple BMP ligands including myostatin, to increase muscle growth. Overall muscle mass did not increase in treated dogs compared to GRMD controls. In keeping with our *GRippet* findings, several indices of motor function declined in treated dogs (Blot S, personal communication, 2015). While these findings are consistent with those of the *GRippets*, they may not have been specific to the effects of myostatin inhibition because a non-selective inhibitor was used.

We have previously shown that myostatin blockade with an AAV8-myostatin propeptide construct administered via regional limb delivery in 3-month-old normal dogs leads to muscle enlargement [31]. As with the *GRippets* reported here, the effects were not uniform among muscles. In particular, the treated VL showed no increase in size, even though transgene levels were comparable to muscles that showed a treatment effect. In a separate study, a group of GRMD dogs were treated systemically with AAV8-myostatin dominant negative propeptide at 10 months of age and followed for 13 months with MRI and at necropsy to determine effects on distal pelvic limb muscle size [32]. All treated GRMD muscles increased in size but the degree varied, ranging from 27 % in the LDE to 49 % in the cranial tibialis. Notably, none of the muscles in either of these studies *decreased* in size. This contrasts with our findings in the *GRIppets*, in which the relative absence of myostatin exaggerated pre-existing trends for either muscle atrophy or hypertrophy. Exaggeration of muscle atrophy, as with the quadriceps, is particularly puzzling and could have considerable potential clinical significance.

Treatments directed at inhibiting myostatin to increase the regenerative response of diseased muscle have been postulated to potentially exhaust satellite cell replicative capacity [24]. Previous studies with dystrophic mouse models and genetic deletion or postnatal inhibition of myostatin are not adequate to address the question of satellite cell senescence since mice have considerably longer telomeres [72] and proportionally higher telomerase activity than humans [73]. Like humans, dogs have relatively short telomeres and low telomerase activity [74, 75]. The *GRippet* is thus a good model to evaluate satellite cell populations in dystrophic muscle with chronic reduction in myostatin. Despite active degeneration and regeneration in *GRippet* muscles, satellite cells were the same proportion (approximately 5–6 %) of myonuclei as in GRMD muscles and did not show evidence of exhaustion.

## Conclusions

Myostatin mRNA and protein levels were down regulated in GRMD dogs independent of myostatin status, suggesting an inherent feedback mechanism intended to promote muscle regeneration. The relative absence of myostatin in the *GRippets* exaggerated pre-existing trends for either muscle atrophy or hypertrophy on MRI. This differential pattern of muscle atrophy/hypertrophy appeared to have functional consequences, in that phenotypic features of the *GRippets* were, if anything, more severe than GRMD dogs with wild-type myostatin. Results from these *GRippet* dogs suggest that dystrophic muscles may be differentially affected by in utero myostatin loss. While data derived from dystrophic dogs lacking myostatin from inception will not necessarily extrapolate to postnatal treatments, this study reinforces the complexity of factors regulating muscle function and further emphasizes that murine models of muscular dystrophy may behave very differently than large animals such as dogs and humans. Although the lack of satellite cell exhaustion in *GRippets* is reassuring for future human experience with myostatin inhibitors, the muscle imbalance and severity of contractures requires continued careful evaluation. These findings may serve to inform future clinical trials of postnatal myostatin inhibition.

## Additional files

Additional file 1:
**Figure S1.** Histograms depicting percent cross-sectional area contributed by each muscle on MRI at midthigh of control, GRMD, and *GRippet* dogs. Pre-existing atrophy or hypertrophy in GRMD muscles is generally more exaggerated in the *GRippets*. (TIF 664 kb) Additional file 2:
**Table S1.** MRI volumes in non-dystrophic control, GRMD (*Mstn* ^*+/+*^), and *GRippet* (*Mstn* ^*+/−*^) Dogs (Mean ± SD). (DOCX 954 kb) Additional file 3:
**Table S2.** T2 mapping values (Mean ± SD) in non-dystrophic control, GRMD (*Mstn* ^*+/+*^), and *GRippet* (*Mstn* ^*+/−*^) Dogs (mean ± SD). (DOCX 369 kb) Additional file 4:
**Table S3.** Histopathologic, morphometric, and molecular findings in non-dystrophic control, GRMD (*Mstn* ^*+/+*^), and *GRippet* (*Mstn* ^*+/−*^) dogs. (DOCX 403 kb) Additional file 5:
**Figure S2.** Pax7-positive cells in *Mstn* ^*+/+*^ and *Mstn* ^*+/−*^ GRMD/*GRippet* and GRMD-carrier dogs. Laminin (A), Pax7 (B), DAPI (C), and merged (D) staining are seen. Satellite cells were defined as Pax7+ nuclei within the laminin + basal lamina (also see data in Additional file 4: Table S3). (TIF 915 kb)

## Abbreviations

AAV: adeno-associated virus
ActRIIB: activin receptor type IIB
ANOVA: analysis of variance
BSA: bovine serum albumin
CK: creatine kinase
CNF: centrally nucleated fibers
CP: circular polarization
CS: cranial sartorius
CSA: cross-sectional area
DAPI: 4',6-diamidino-2-phenylindole
DIC: differential interference contrast
DMD: Duchenne muscular dystrophy
DNA: deoxyribonucleic acid
DSHB: Developmental Studies Hybridoma Bank
DTT: dithiothreitol
dy(W): laminin alpha2-deficient mouse
Dyf^−/−^: mouse model of LGMD2B and Myoshi myopathy
ECD: eccentric contraction deficit
ECL: enhanced chemilumescent
EDL: extensor digitorum longus
Fd: force deficit
FDR: false discovery rate
GDF8: growth and differentiation factor 8
GRMD: golden retriever muscular dystrophy
H&E: hematoxylin and eosin
HP: hydroxyproline
HPRT1: hypoxanthine phosphoribosyltransferase 1
HSD: honest significant differences
LC-MS: liquid chromatography-mass spectrometry
LC-MS-MS: liquid chromatography-mass spectrometry and Liquid chromatography-tandem mass spectrometry
LHG: lateral head of gastrocnemius
MRI: magnetic resonance imaging
mRNA: messenger ribonucleic acid
MSE-T2: multi-spin-echo T2
Mstn: myostatin
NIH: National Institutes of Health
Pax7: paired box 7
PBST: phosphate buffered saline containing 0.05 % Tween-20
PCR: polymerase chain reaction
Po: maximal isometric tetanic force
PVDF: polyvinylidene difluoride
qRT-PCR: quantitative real-time polymerase chain reaction
RNA: ribonucleic acid
ROM: range of motion
T_2_fs: T_2_-weighted image sequences with fat saturation
T_2_w: T_2_-weighted image sequences without fat saturation
TPER: tissue protein extraction reagent
TSE: turbo spin echo
TTJ: tibiotarsal joint
UNC-CH: University of North Carolina-Chapel Hill
VL: vastus lateralis
