# Supplementary figures and images for: Dystrophin-deficient dogs with reduced myostatin have unequal muscle growth and greater joint contractures
Source: Skelet Muscle. 2016 Apr 4;6:14. doi: 10.1186/s13395-016-0085-7 (PMC4819282; doi:10.1186/s13395-016-0085-7)

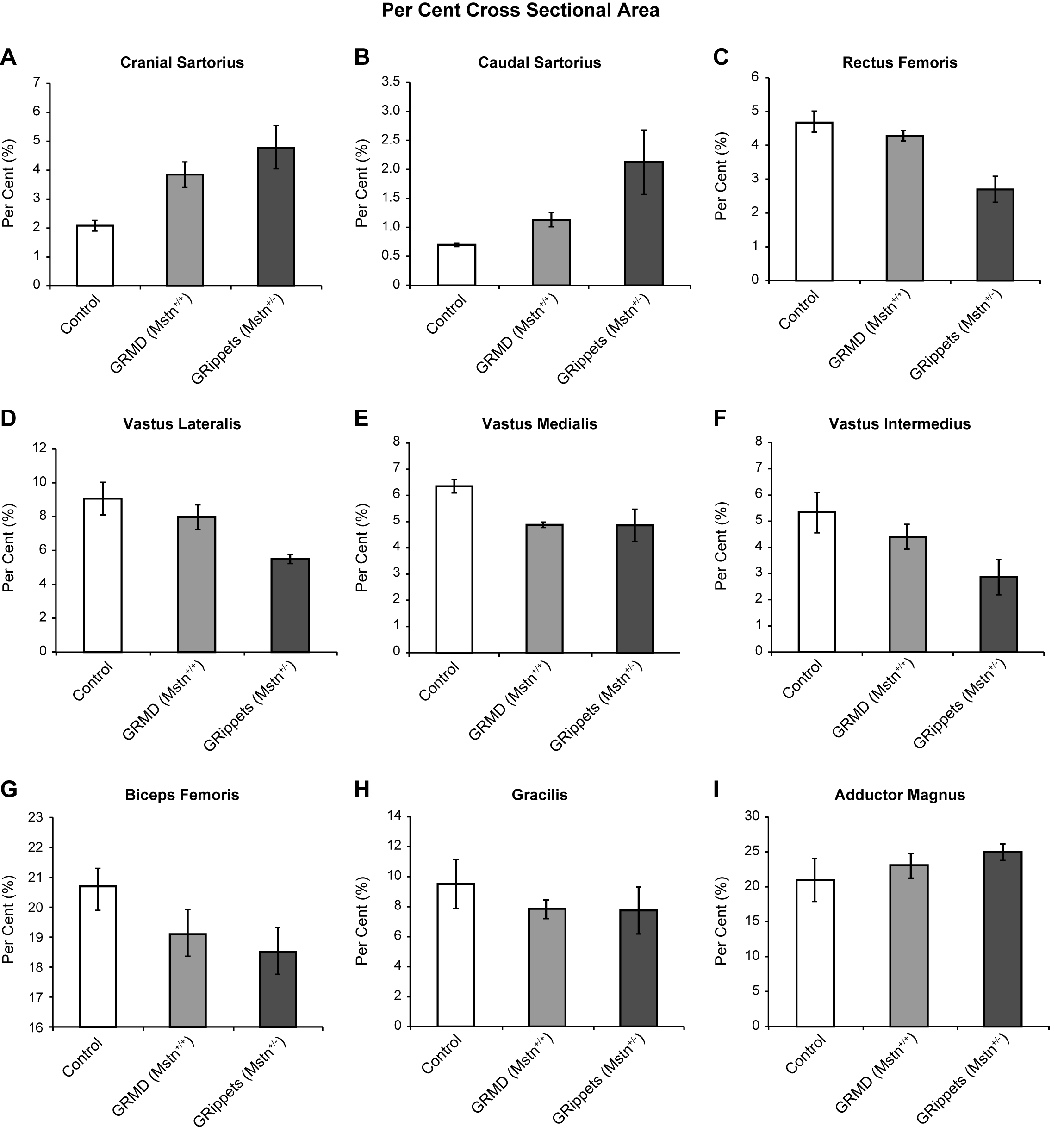

Supplement: Additional file 1: — Figure S1. Histograms depicting percent cross-sectional area contributed by each muscle on MRI at midthigh of control, GRMD, and GRippet dogs. Pre-existing atrophy or hypertrophy in GRMD muscles is generally more exaggerated in the GRippets. (TIF 664 kb) [file 13395_2016_85_MOESM1_ESM.tif]

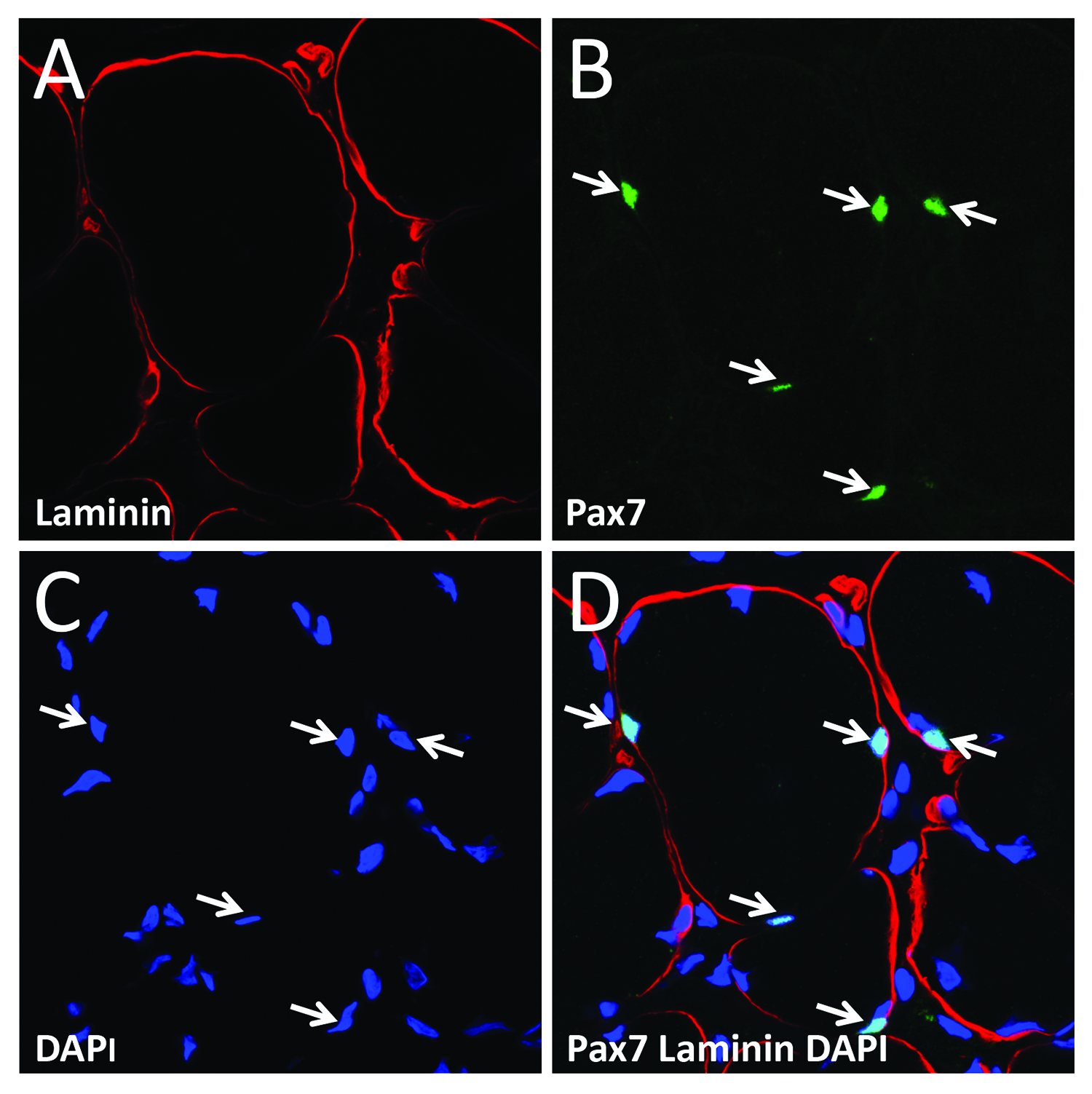

Supplement: Additional file 5: — Figure S2. Pax7-positive cells in Mstn +/+ and Mstn +/− GRMD/GRippet and GRMD-carrier dogs. Laminin (A), Pax7 (B), DAPI (C), and merged (D) staining are seen. Satellite cells were defined as Pax7+ nuclei within the laminin + basal lamina (also see data in Additional file 4: Table S3). (TIF 915 kb) [file 13395_2016_85_MOESM5_ESM.tif]
