# Supplementary material for: Dystrophin-deficient dogs with reduced myostatin have unequal muscle growth and greater joint contractures
Source: Skelet Muscle. 2016 Apr 4;6:14. doi: 10.1186/s13395-016-0085-7 (PMC4819282; doi:10.1186/s13395-016-0085-7)
Supplement: Additional file 2: — Table S1. MRI volumes in non-dystrophic control, GRMD (Mstn +/+), and GRippet (Mstn +/−) Dogs (Mean ± SD). (DOCX 954 kb) [file 13395_2016_85_MOESM2_ESM.docx]

| **Supplemental Table 1. MRI Volumes in Non-dystrophic Control, GRMD (*Mstn+/+*), and *GRippet* (*Mstn+/-*) Dogs (Mean ± SD)** | | | | | | | | | |
| --- | --- | --- | --- | --- | --- | --- | --- | --- | --- |
|  | **Non-Dystrophic Controls (n = 3)** | | | **GRMD (*Mstn^+/+^*) (n = 3)** | | | ***GRippets* (*Mstn^+/-^*) (n = 4)** | | |
| **Muscle** | **Volume**  **(mm^3^)** | **Volume/Femur Length (mm^3^/mm)** | **Percent Cross Section** | **Volume**  **(mm^3^)** | **Volume/Femur Length (mm^3^/mm)** | **Percent Cross Section** | **Volume**  **(mm^3^)** | **Volume/Femur**  **Length**  **(mm^3^/mm)** | **Percent Cross**  **Section** |
| Cranial Sartorius | 617±110 | 3.69±0.66 | 2.08±0.36 | 754±157 | 4.76±0.90 | 3.85±0.87 | 1,036±312 | 6.52±1.92 | 4.77±1.50 |
| Caudal Sartorius | 208±19.5 | 1.25±0.17 | 0.70±0.06 | 224±60.5 | 1.41±0.37 | 1.13±0.25 | 460±219 | 2.88±1.36 | 2.13±1.10 |
| **Sartorius Total** | **825±126** | **4.95±0.79** | **2.78±0.41** | **978±182** | **6.17±1.02** | **4.98±0.91** | **1,496±519** | **9.40±3.19** | **6.90±2.56** |
| Rectus Femoris | 1,387±188^a*,b**^ | 8.30±1.07^a*,b**^ | 4.68±0.62 | 844±68.3 | 5.34±0.34 | 4.29±0.30 | 592±181 | 3.71±1.05 | 2.70±0.76 |
| Vastus Lateralis | 2,684±546^a*,b**^ | 16.0±2.15^a*,b**^ | 9.07±1.93 | 1,569±304 | 9.90±1.71 | 7.98±1.46 | 1,198±134 | 7.53±0.69 | 5.50±0.53 |
| Vastus Medialis | 1,882±164^a**,b**^ | 11.3±1.48^a*,b*^ | 6.35±0.50 | 959±28.0 | 6.07±0.20 | 4.88±0.20 | 1,048±226 | 6.64±1.65 | 4.86±1.22 |
| Vastus Intermedius | 1,579±440^b*^ | 9.38±2.17^b*^ | 5.34±1.54 | 868±218 | 5.51±1.51 | 4.39±0.95 | 637±340 | 4.00±2.07 | 2.87±1.35 |
| **Quadriceps Total** | **7,533±822^a**,b**^** | **44.9±1.84^a***,^**^b***^ | **25.4±2.99^b**^** | **4,240±324** | **26.8±1.84** | **21.6±0.94** | **3,475±312** | **21.9±2.00** | **15.9±0.61** |
| Semimembranosus | 4,157±979 | 25.1±7.30 | 14.0±3.30 | 2,633±356 | 16.7±2.53 | 13.4±1.15 | 3,619±623 | 22.9±5.20 | 16.7±3.44 |
| Semitendinosus | 1,946±43.1 | 11.7±0.74 | 6.57±0.20 | 2,001±1,014 | 12.6±6.17 | 10.0±4.66 | 1,974±586 | 12.4±3.63 | 9.16±3.20 |
| **Semimembranosus/Semitendinosus Total** | **6,103±999** | **36.8±7.99** | **20.6±3.38** | **4,634±1,157** | **29.3±7.03** | **23.4±4.64** | **5,592±587** | **35.4±5.21** | **25.9±4.31** |
| Biceps Femoris | 6,138±415^a**,b**^ | 36.7±2.14^a*,b**^ | 20.7±1.40 | 3,761±355 | 23.8±2.80 | 19.1±1.56 | 4,047±501 | 25.5±3.28 | 18.5±1.57 |
| Gracilis | 2,806±940 | 16.7±5.17 | 9.49±3.25 | 1,543±266 | 9.79±1.92 | 7.84±1.25 | 1,714±775 | 10.8±4.69 | 7.73±3.12 |
| Adductor Magnus | 6,225±1,878 | 37.6±12.4 | 21.0±6.16 | 4,526±473 | 28.7±3.44 | 23.1±3.54 | 5,467±759 | 34.3±3.15 | 25.0±2.36 |
| CS/VL | 0.24±0.06^b**^ | 0.24±0.0^b**^ | 0.24±0.06^b*^ | 0.48±0.04 | 0.48±0.04 | 0.48±0.04 | 0.86±0.19 | 0.86±0.19 | 0.86±0.19 |
| **TOTAL** | **29,631±274^a**,b**^** | **178±13.4^a**,^** ^b**^ | **100.00** | **19,682±1,255** | **125±8.87** | **100.00** | **21,791±1,409** | **137±7.76** | **100.00** |

^a^Significantly different (P < 0.05^*^; < 0.01^**^; < 0.001^***^) from GRMD dogs.

^b^Significantly different (P < 0.05^*^; < 0.01^**^; < 0.001^***^) from *GRippet*s.
