# Supplementary material for: Dystrophin-deficient dogs with reduced myostatin have unequal muscle growth and greater joint contractures
Source: Skelet Muscle. 2016 Apr 4;6:14. doi: 10.1186/s13395-016-0085-7 (PMC4819282; doi:10.1186/s13395-016-0085-7)
Supplement: Additional file 3: — Table S2. T2 mapping values (Mean ± SD) in non-dystrophic control, GRMD (Mstn +/+), and GRippet (Mstn +/−) Dogs (mean ± SD). (DOCX 369 kb) [file 13395_2016_85_MOESM3_ESM.docx]

| **Supplemental Table 2. T2 Mapping Values (Mean±SD) in Non-dystrophic Control,**  **GRMD (*Mstn^+/+^*), and *GRippet* (*Mstn^+/-^*) Dogs (Mean ± SD)** | | | |
| --- | --- | --- | --- |
| **Muscle** | **T2 Mapping Value** | | |
|  | Controls | GRMD (*Mstn^+/+^*) | *GRippet* (*Mstn^+/-^*) |
| Cranial Sartorius | 49.6±2.10 | 52.6±5.27 | 45.6±1.56 |
| Caudal Sartorius | 53.1±6.22 | 66.8±4.05 | 58.5±7.15 |
| **Total Sartorius** | **50.6±2.40** | **56.1±3.36** | **49.2±1.04** |
| Rectus Femoris | 36.8±3.08^b*^ | 49.9±6.35 | 51.7±5.64 |
| Vastus Lateralis | 38.1±5.00 | 52.6±6.73 | 51.3±4.92 |
| Vastus Medialis | 39.9±5.26^b*^ | 54.8±9.70 | 56.7±4.66 |
| Vastus Intermedius | 38.6±5.32 | 49.6±8.98 | 54.8±6.41 |
| **Quadriceps Total** | **38.5±4.70**^b*^ | **51.88±7.75** | **53.7±4.47** |
| Semimembranosus | 36.7±5.23^a*,b*^ | 52.8±5.02 | 53.2±5.54 |
| Semitendinosus | 38.5±6.01^b*^ | 50.6±5.38 | 53.2±2.63 |
| **Semimembranosus/**  **Semitendinosus Total** | **37.3±5.50^a*,b*^** | **51.9±4.72** | **53.3±4.38** |
| Biceps Femoris | 36.7±4.65^a*b*^ | 50.1±4.53 | 50.9±2.36 |
| Gracilis | 41.0±5.80^a*,b*^ | 57.4±5.23 | 56.7±3.53 |
| Adductor | 37.2±4.96^b*^ | 50.5±4.45 | 50.6±5.16 |
| CS/VL | 1.31±0.11^b*^ | 1.01±0.15 | 0.89±0.08 |
| **Weighted Mean ± SD** | **38.2±5.00 ^a*,b*^** | **51.9±5.09** | **52.3±3.47** |

^a^Significantly different (P < 0.05^*^; < 0.01^**^) from GRMD dogs.

^b^Significantly different (P < 0.05^*^; < 0.01^**^) from *GRippet*s.
