# Supplementary material for: Dystrophin-deficient dogs with reduced myostatin have unequal muscle growth and greater joint contractures
Source: Skelet Muscle. 2016 Apr 4;6:14. doi: 10.1186/s13395-016-0085-7 (PMC4819282; doi:10.1186/s13395-016-0085-7)
Supplement: Additional file 4: — Table S3. Histopathologic, morphometric, and molecular findings in non-dystrophic control, GRMD (Mstn +/+), and GRippet (Mstn +/−) dogs. (DOCX 403 kb) [file 13395_2016_85_MOESM4_ESM.docx]

| **Supplemental Table 3. Histopathologic, Morphometric, and Molecular Findings in Non-dystrophic Control, GRMD (*Mstn^+/+^*),**  **and *GRippet* (*Mstn^+/-^*) Dogs** | | | | | | | | | |
| --- | --- | --- | --- | --- | --- | --- | --- | --- | --- |
| Group | Muscle | mRNA  (fold change) | Protein | Degenerating Fibers  (%; mean±SD) | Regenerating Fibers  (%; mean±SD) | CNF  (%; mean±SD) | CSA  (mμ^2^; mean±SD) | HP  (μg/mg protein; mean±SD) | ActRIIB (arbitrary units; mean±SD) |
| Controls^#^ | Average | 1.06±0.09 | 7.63±3.51 | 0.17±0.30^a**,b**^ | 0.26±0.40 | 1.06±0.95^a*,b**^ | 2,842±429^a*^ | 9.53±2.56^a*^ | 0.22±0.06 |
| GRMD (*Mstn^+/+^*) | Average | 0.51±0.50 | 4.30±0.98 | 3.97±1.21 | 1.72±1.20 | 9.74±4.19 | 2,095±155 | 26.5±9.30 | 1.06±0.32 |
| *GRippets* (*Mstn^+/-^)* | Average | 0.60±0.27 | 2.94±1.48 | 2.58±0.36 | 0.90±0.44 | 12.3±2.82 | 2,730±211 | 22.9±4.79 | 0.92±0.52 |
| Controls^#^ | CS | 1.08±0.46^a**, b**^ | 7.79±2.18^a*,b*^ | 0.52±0.91 | 0.07±0.12 | 0.99±0.39^a*,b**^ | 2,598±346^b**^ | 10.1±5.00 | 0.13±0.08 |
|  | LDE | 1.08±0.56 | 8.68±6.16 | 0.00±0.00^a*^ | 0.00±0.00 | 1.34±1.70^a*,b*^ | 2,898±643 | 13.0±7.57 | 0.25±0.22 |
|  | VL | 1.05±0.39^a*, b*^ | NE | 0.00±0.00 | 0.58±1.00 | 0.86±0.87^a*,b*^ | 3,196±326^a*,b**^ | 7.20±1.63 | 0.35±0.07 |
|  | LHG | 1.02±0.22 | 6.42±2.20 | 0.16±0.28 | 0.39±0.51 | 1.07±1.52 | 2,677±745 | 7.80±0.75 | 0.14±0.18 |
| GRMD/*Mstn^+/+^* | CS | 0.09±0.06 | 1.63±0.39 | 4.09±2.38 | 0.76±0.66 | 11.4±5.23 | 2,661±316^b**^ | 34.5±12.0 | 1.53±0.34 |
|  | LDE | 1.06±1.53 | 5.21±3.71 | 5.97±2.81 | 3.56±3.95 | 13.0±6.73 | 2,007±217 | 16.3±1.35 | 1.12±0.55 |
|  | VL | 0.31±0.20 | NE | 2.89±1.76 | 0.59±0.53 | 4.39±1.27 | 1,850±610 | 18.5±6.18 | 1.33±0.59 |
|  | LHG | 0.56±0.45 | 6.07±1.16 | 2.95±2.48 | 1.99±1.87 | 10.2±6.02 | 1,864±466 | 36.9±20.9 | 0.25±0.15 |
| *GRippet*/*Mstn^+/-^* | CS | 0.04±0.03 | 0.92±0.73 | 3.92±1.02 | 0.43±0.46 | 20.7±3.82 | 4,133±233^a**^ | 19.3±8.89 | 0.99±0.49 |
|  | LDE | 0.69±0.55 | 4.43±3.14 | 2.39±1.01 | 1.32±1.22 | 13.4±2.11 | 2,176±553 | 30.9±16.6 | 1.42±0.94 |
|  | VL | 0.34±0.19 | NE | 2.18±0.85 | 0.66±0.59 | 4.55±1.47 | 1,560±155 | 22.3±6.3 | 1.07±0.61 |
|  | LHG | 1.35±0.68 | 3.45±1.59 | 1.82±1.06 | 1.20±1.04 | 10.5±5.30 | 1,613±340 | 19.1±17.1 | 0.21±0.14 |

CNF = Central nucleated fibers; HP = Hydroxyproline; CSA = Cross sectional area; ActRIIB = Activin receptor type IIB; NE = Not evaluated.

^$^Myostatin protein data was assessed in the CS, LDE, and LHG from eight dogs: *GRippets* Dash, Derwood, and Abner; GRMD Flash and Samantha; and controls Racer and Esmerelda.  Samples of the VL were no longer available.

^#^The control group for mRNA, DF, RF, CNF, and HP includes *Racer*, *Endora*, and *Esmeralda*; for ActRIIB, only *Endora* and *Racer* are included.

^a^Significantly different (P < 0.05^*^; < 0.01^**^) from GRMD dogs.

^b^Significantly different (P < 0.05^*^; < 0.01^**^) from *GRippet*s.
